# Analysis of cell-type-specific chromatin modifications and gene expression in *Drosophila* neurons that direct reproductive behavior

**DOI:** 10.1371/journal.pgen.1009240

**Published:** 2021-04-26

**Authors:** Colleen M. Palmateer, Shawn C. Moseley, Surjyendu Ray, Savannah G. Brovero, Michelle N. Arbeitman

**Affiliations:** 1 Department of Biomedical Sciences, Florida State University, College of Medicine, Tallahassee, Florida, United States of America; 2 Program of Neuroscience, Florida State University, Tallahassee, Florida, United States of America; Katholieke Universiteit Leuven, BELGIUM

## Abstract

Examining the role of chromatin modifications and gene expression in neurons is critical for understanding how the potential for behaviors are established and maintained. We investigate this question by examining *Drosophila melanogaster fru P1* neurons that underlie reproductive behaviors in both sexes. We developed a method to purify cell-type-specific chromatin (Chromatag), using a tagged histone H2B variant that is expressed using the versatile Gal4/UAS gene expression system. Here, we use Chromatag to evaluate five chromatin modifications, at three life stages in both sexes. We find substantial changes in chromatin modification profiles across development and fewer differences between males and females. Additionally, we find chromatin modifications that persist in different sets of genes from pupal to adult stages, which may point to genes important for cell fate determination in *fru P1* neurons. We generated cell-type-specific RNA-seq data sets, using translating ribosome affinity purification (TRAP). We identify actively translated genes in *fru P1* neurons, revealing novel stage- and sex-differences in gene expression. We also find chromatin modification enrichment patterns that are associated with gene expression. Next, we use the chromatin modification data to identify cell-type-specific super-enhancer-containing genes. We show that genes with super-enhancers in *fru P1* neurons differ across development and between the sexes. We validated that a set of genes are expressed in *fru P1* neurons, which were chosen based on having a super-enhancer and TRAP-enriched expression in *fru P1* neurons.

## Author summary

Differences in male and female reproductive behaviors are pervasive in nature and important for species propagation. Studies of sex differences in the fruit fly, *Drosophila melanogaster*, have been ongoing since the early 1900s, with many of the critical molecular and neural circuit determinates that create sexually dimorphic behavior identified. This system is a powerful model to understand fundamental principles about the underpinnings of complex behavior at high resolution. In this study, we examine the gene expression and chromatin modification differences in a set of neurons that direct male and female reproductive behaviors in *Drosophila*. We describe differences across development and between the sexes with the goal of understanding how the potential for behavior is created and maintained.

## Introduction

Innate reproductive behaviors are directed by neurons that attain their cell fate during development. Cell fate identity is then maintained during adult stages for the performance of behavior. For neurons, plasticity and modification by experience are an intrinsic part of their function. Chromatin and DNA modifications have been implicated as a mechanism that could direct both long-lasting cell fate identity and allow for plasticity, by directing cell-type-specific transcriptional programs that can be modified in response to the environment. Indeed, studies have found chromatin and DNA modifications play a role in synaptic plasticity, learning, memory, stress response, and complex social behaviors across phyla [1–5]. Furthermore, DNA and chromatin modifications can provide a mechanism to direct stage-specific and sex-differences in behavior, by overlaying time- and sex-specific information onto the genome that is less transient than transcriptional changes. Here, we examine neurons underlying *Drosophila melanogaster* reproductive behaviors to investigate the dynamic developmental and sex-specific roles of chromatin modifications for altering gene expression in neurons that direct complex, innate behaviors.

**Fig 1:**
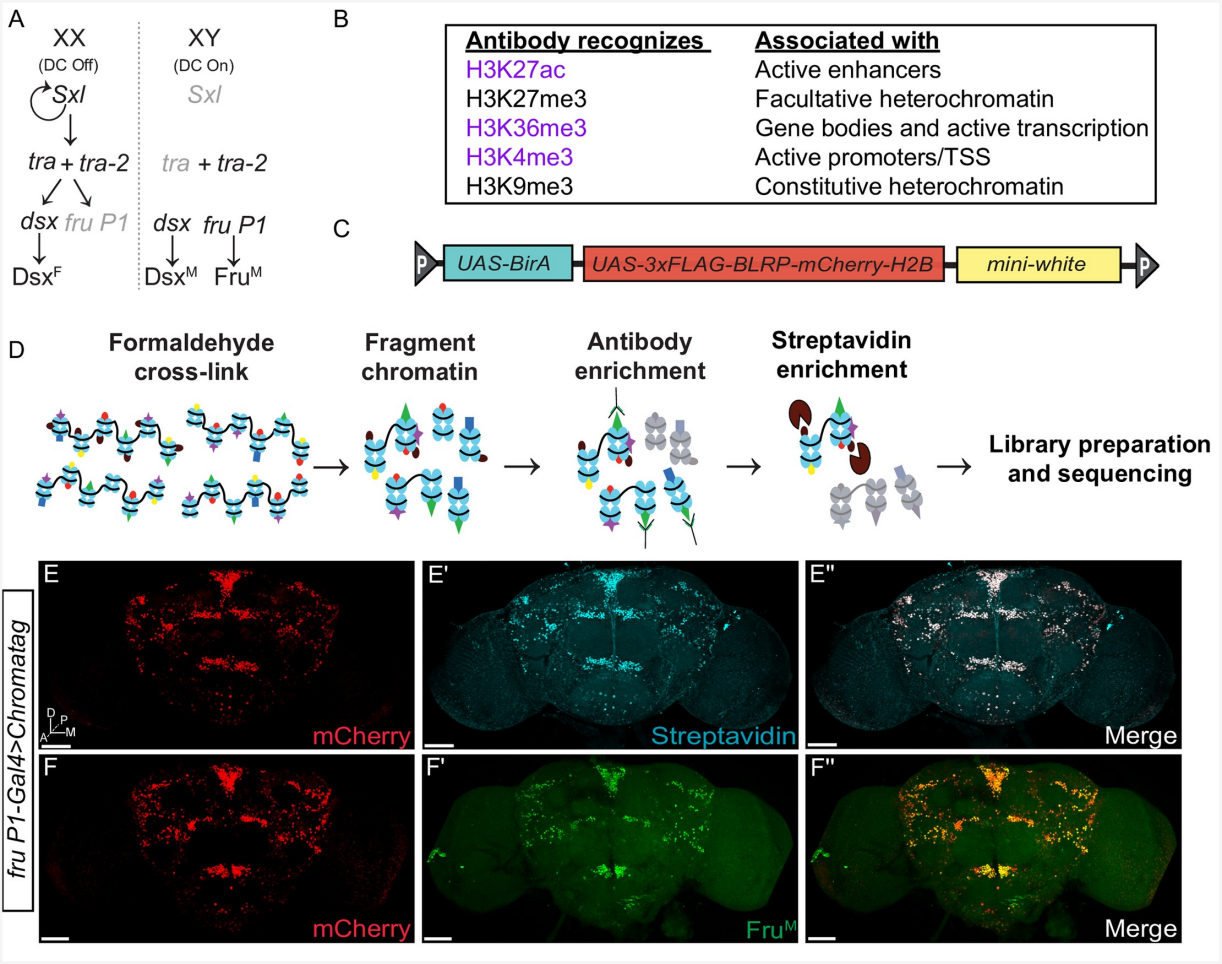
Chromatag methodology enables cell-type-specific ChIP in *fru P1* neurons. **(A)** The *Drosophila* somatic sex determination hierarchy is composed of an alternative pre-mRNA splicing cascade dependent on the number of X chromosomes. Alternative pre-mRNA splicing of *doublesex* (*dsx*) and *fruitless* (*fru P1*) products generates sex-specific transcription factors: Dsx^F^, Dsx^M^, and Fru^M^. Sex-specific Dsx and Fru regulate gene expression to yield sex differences in morphology, neuroanatomy and ultimately behavior [6,7,89] **(B)** Histone H3 modifications profiled in this study and their role in transcriptional activation (purple) or repression (black) [36–40]. Abbreviation: transcriptional start site (TSS). **(C)** Schematic of the *Chromatag* transgene. The construct includes three transgenes. One directs expression of amino-terminal tagged H2B under UAS control; the tag contains 3x-FLAG, the Biotin Ligase Recognition Peptide (BLRP) and *mCherry*. Another transgene directs biotin ligase (*BirA*) expression under UAS control. The *mini-white* transgene is a germline transformation marker. **(D)** Overview of the Chromotag-ChIP workflow. Tissue from flies expressing Chromatag is fixed with formaldehyde, the chromatin is isolated and then fragmented by sonication. An antibody to a histone modification is used in the first ChIP. A subsequent pull-down with streptavidin-bound beads enriches for cell-type-specific chromatin. The purified genomic DNA undergoes library preparation and sequencing. **(E-F”)** Immunostaining of 10–12 day adult male brains expressing the *Chromatag* transgene in *fru P1* neurons. **(E-E”)** The Chromatag H2B variant is visualized by the mCherry tag (red) and overlaps with biotinylated H2B detected using fluorescently conjugated streptavidin (cyan). **(F-F”)** The Chromatag H2B variant is visualized by the mCherry tag (red) and overlaps with Fru^M^ based on anti-Fru^M^ immunostaining (green). Three-dimensional confocal maximum projections from successive confocal slices are presented, with the most anterior slice positioned first. Brain anatomical axes are presented on lower left of first brain image panel (see **E**): D, *dorsal*; M, *medial*; A, *anterior*; P, *posterior*. For *Chromatag* transgene expression at other time points in this study see **S1 Fig**. Further information on the *Drosophila* central nervous system, including anatomical structures and nomenclature, has been previously described [90,106]. **(E-F”)** Flies are the genotype: *w/Y; P[w*^*+mC*^, *UAS-GAL4]/P[w*^*+mC*^, *UAS-Chromatag]; fru P1-Gal4/+*. Scale bars = 50 μm.

Reproductive behaviors in *Drosophila* include a multi-step, male courtship display towards the female [6,7]. If the female chooses to mate, she will slow down to allow mating to occur and subsequently will display post-mating behaviors that include egg-laying, and additional post-mating changes [8,9]. The potential for these sexually dimorphic behaviors is specified by the sex hierarchy transcription factors encoded by *doublesex* (*dsx*) and *fruitless* (*fru*) [10–15] (**Fig 1A**). Sex-specific Dsx and Fru are produced in subsets of neurons important for reproductive behaviors (< 5% of neurons). These transcription factors are first detected in the nervous system in late third instar larvae when the nervous system begins to be remodeled for adult function, are present throughout metamorphosis, and persist into adult stages [14–20].

*fru* was first identified as important for reproductive behavior based on mutants that had altered male courtship behaviors [21]. In-depth, molecular-genetic analysis showed that *fru* is a complex locus containing at least four promoters (P1-P4), with the *fru P1* promoter driving expression of a transcript class (*fru P1*) that is spliced downstream of the sex determination hierarchy (**Fig 1A)** [11,12]. Sex-specific splicing of *fru P1* transcripts results in production of male-specific transcription factor isoforms (Fru^M^) that are members of the BTB family, whereas *fru P1* transcripts in females have an early stop codon, with a coding potential of 94 amino acids of unknown function [11,12]. Fru^M^ isoforms contain an additional amino-terminal 101 amino acid region with no known function, differentiating them from common Fru isoforms produced by P2-P4 promoters [11,12]. Mutations impacting *fru P1* transcripts alter several aspects of male courtship, but do not appear to alter female behaviors, though some allele combinations showed female sterility [11,12,22–24]. Additionally, forcing male-specific splicing or producing Fru^M^ in females allows for some male courtship behaviors in females [19,25]. However, even though *fru P1* products have a major role in only male behavior, *fru P1*-expressing neurons (*fru P1* neurons hereafter) are present in both sexes and direct the distinct male and female reproductive behaviors [19,20,25,26]. A major remaining question is how does a largely shared *fru P1* neuroanatomical substrate and shared genome give rise to different male and female reproductive behaviors.

It is clear that *fru P1* neurons and *fru P1* expression are modified during both developmental and adult stages. Sex differences in the number and morphology of some *fru P1* neurons have been shown to be established during metamorphosis [17,20,27]. Furthermore, early genetic studies using a temperature-sensitive allele of the sex hierarchy gene *transformer 2*, which regulates splicing of *fru P1*, showed that the middle of metamorphosis was a critical period for establishing sex-specific behaviors, and also showed that there was adult plasticity in terms of behavioral potential [28]. Later studies, performed by overexpressing the female-splice-form of the sex hierarchy gene *transformer* (*tra*), which also regulates splicing of *fru P1*, showed that there is an irreversible period critical for establishing the potential for sex-specific behavior during early metamorphosis [29]. In the adult, *fru P1* expression is regulated in *Or47b* and *Ir84a* olfactory receptor neurons (ORNs), and this is dependent on adult olfactory activity [30]. Additionally, an increase in adult *fru P1* expression in *Or47b* ORNs has been linked to an increase in activating chromatin modifications at the *fru P1* promotor [31]. Further, *fru P1* expression in adult *Or47b* ORNs is required for increased copulation rate in group housed males compared to single housed males [32]. These studies point to developmental and adult stages as critical for establishing the potential for behavior. Therefore, an examination of the molecular-genetic basis of reproductive behavior directed by *fru P1* should include understanding of both developmental and adult stages.

One mechanism to generate and maintain behavioral potential is through chromatin modifications. A previous study demonstrated that Fru^M^ forms a complex with transcriptional cofactor, Bonus, a TIF1 homolog [33]. This Fru^M^-Bonus complex selectively recruits the chromatin modifying protein Histone deacetylase 1 (HDAC1/Rpd3) or Heterochromatin protein 1a (HP1a/Su(var)205) [33]. If the Fru^M^-Bonus complex recruits HDAC1, a set of sexually dimorphic interneurons (mAL) are masculinized, whereas recruitment of HP1a results in demasculinized morphology of the mAL neurons [33]. This suggests that differences in chromatin modifications in *fru P1* neurons may contribute to gene expression changes. Here, we ask about differences in the chromatin landscape in male and female *fru P1* neurons, how chromatin modifications change across developmental and adult stages, and if chromatin modifications are a mechanism to direct stage- and sex-specific gene expression that direct behavior.

To determine the chromatin landscape in *fru P1* neurons, we developed a transgenic strategy to purify chromatin using a tagged histone H2B, under Gal4/UAS transcriptional control (called Chromatag). Complementary to other cell-type-specific chromatin purification methods, Chromatag allows for the direct purification of chromatin and profiling of chromatin modifications without requiring fluorescence-activated cell sorting (FACS) purification [34,35]. Using this method, we purified chromatin from *fru P1* neurons of both sexes and examine five chromatin modifications (H3K27ac, H3K36me3, H3K4me3, H3K27me3, and H3K9me3), with conserved roles in regulating gene expression (**Fig 1B**) [36–40].We examine chromatin modifications at three stages: mid-metamorphosis at 48 hours after puparium formation (48hr APF), 1-day adults (frozen 16–24 hrs post-eclosion), and 10-12-day adults, to gain insight into how the potential for behavior is established and maintained. In addition, we examined chromatin modifications in all neurons within the head (*elav*-*Gal4*) in 1-day adults to compare with neurons from 1-day adult *fru P1* neurons. First, we determined the positions of histone modification peaks in genic regions and whether they persist from pupal to adult stages or are life-stage-specific modifications. Using hierarchical clustering, we examined the combinations of histone modifications on all annotated genes and observe greater changes in profiles across developmental stages than between sexes. Additionally, we identified actively translated mRNAs in *fru P1* neurons using translating ribosome affinity purification (TRAP), which revealed stage- and sex-specific gene expression. To assess if different combinations of H3 modifications are correlated with transcriptional activity in *fru P1* neurons, we perform a meta-analysis with data from cell-type-specific transcriptional studies (TRAP) [41,42]. We find that a subset of histone modification patterns is characteristic of expressed genes but that no pattern is indicative of high expression. Using our H3K27ac modification peaks, we have identified super-enhancer (SE) regions and show that they are largely stage specific. We chose a set of SE-containing genes that were also identified in our *fru P1* TRAP data sets and confirm that they are expressed in *fru P1* neurons. Altogether, we discovered critical chromatin modifications and gene expression patterns that are time point and sex-specific that underlie the potential for complex behavior.

## Results

### Cell-type-specific strategy to analyze chromatin modifications

To examine the chromatin modification landscape in a cell-type-specific manner we developed transgenic strains to express tagged histone H2B under Gal4/UAS control [43]. This approach is versatile and can be used with other Gal4 drivers to purify chromatin from different cell-types [44]. The transgene contains a cDNA, under UAS control, for expression of H2B tagged with amino-terminal 3xFLAG, biotin ligase recognition peptide (BLRP), and mCherry (Chromatag; **Fig 1C**, red). The transgene also contains the *E*. *coli* biotin ligase gene, *birA*, under UAS control [45] (**Fig 1C**, blue). To perform cell-type-specific ChIP-seq, a sequential, two-step chromatin immunoprecipitation procedure was used, where we first enriched for chromatin fragments containing a histone modification, using an antibody purification, and then enriched for chromatin fragments from the cell-type of interest, using streptavidin purification (**Fig 1D**).

We first validated that the transgene is expressed in a cell-type-specific manner, by examining *UAS*-*Chromatag* expression driven by *fru P1-Gal4* (*fru P1-Gal4>Chromatag*). We show that mCherry-tagged H2B overlaps with Fru^M^ immunostaining, and that fluor-conjugated streptavidin staining is restricted to cells where mCherry-H2B is detected (**Fig 1E and 1F**), at all time points examined (**S1 Fig**). In addition, we do not detect mCherry-tagged H2B in the central nervous system in the absence of a Gal4 driver (**S1B–S1G” Fig**). To check the versatility of this approach, we tested the viability of flies with ubiquitous *Chromatag* expression, by driving expression with *daughterless*-*Gal4* driver (*da-Gal4*). We find that male and female *da-Gal4>Chromatag* flies are viable (**S2A Fig**). To confirm that Chromatag can be incorporated throughout the genome, we performed sequential ChIP-seq with an antibody to histone H3. As expected, we observed high coverage across all chromosomes (78–91% coverage), at all time points and in both cell-types examined (**S1 Table**). It is not possible to uniquely align short-read ChIP-seq data to repetitive sequence regions, which accounts for not having closer to 100% coverage. For example, we observed the lowest coverage on the 4^th^ chromosome which has highly repetitive DNA sequence content [46,47].

To determine if there are behavioral impacts of producing Chromatag in neurons, we examined the locomotor activity of *fru P1-Gal4>Chromatag* flies. There are no significant differences in locomotor activity between single transgene control flies and *fru P1-Gal4>Chromatag* flies (**S2B Fig**). Additionally, we assayed courtship behavior in *fru P1-Gal4>Chromatag* flies. We find that *fru P1-Gal4>Chromatag* males court females normally and are similar to single transgene controls (**S2C–S2F Fig**). We do find elevated male-male courtship in *fru P1-Gal4>Chromatag* flies, compared to single transgene controls, which is often seen with transgenic strains harboring the mini *white* cDNA, used as a marker for germline transformation (**S2G–S2I Fig**) [48–51].

### Correlation across chromatin modification data sets

In this study we generated ChIP-seq data sets for five histone H3 modifications in *fru P1* and *elav* neurons from both sexes (**Figs 1B** and S1A). We examined three activating modifications (H3K27ac, H3K4me3 and H3K36me3), and two repressive modifications (H3K27me3 and H3K9me3), with 3–4 biological replicates for each stage and sex. Using the deepTools package, ChIP-seq read count signals for each data set were normalized to the matched ChIP-seq input libraries in 50bp bins and the log2 ratio of signal was calculated [52]. We next used the Spearman correlation heatmap function in deepTools to evaluate the positional correlation of genome-wide ChIP-seq signals from all the data sets comparing 10kb bins (**S3 Fig**), with color indicating correlation. The results show that the data sets for the same H3 histone modification are nearly all positively correlated (**S3 Fig**, blue regions in heatmap). The datasets for the two repressive modifications (H3K9me3 and H3K27me3) cluster together (pink highlighted node in dendrogram), as might be expected given these modifications are known to decorate heterochromatic regions [36,37]. The strongest anti-correlation occurs between the samples from adult stages for H3K27ac and H3K36me3 (light blue and dark blue nodes in dendrogram), indicating that these modifications are occurring in different gene regions and/or genes. The anti-correlation of H3K27ac and H3K36me3 is also consistent with H3K27ac residing in promotor regions and H3K36me3 in gene bodies [53,54]. Additionally, these results show that the Chromatag approach is effective in cell-type-specific chromatin enrichment, given that the male and female data sets for each cell-type (*elav* and *fru P1*) are highly correlated with each other, in a stage-specific manner. Furthermore, the correlation analysis provides validation, as data for the same chromatin modification largely cluster together and show the cell-type specificity of the Chromatag approach. This analysis indicates that the distribution for each chromatin modification shows time point, cell-type and sex differences.

### An examination of chromatin modification peaks in cell-types and across time points

Next, we used the MACS2 peak calling algorithm to identify genome regions enriched for each chromatin modification. We called peaks using pooled biological replicate data, as well as on individual replicates (**S1–S10 Data**). We define consensus peaks as those occurring in at least 50% of individual replicates (**S1–S10 Data**) [55]. We limited our analysis to peaks that are within three kilobases (kb) of an annotated gene, resulting in 16,385 genes containing at least one peak, across the five modifications (**S1 Table**). We note that even though we are able to effectively ChIP and sequence DNA from the male X chromosome, the MACS2 peak calling algorithm called very few peaks for the male X chromosome (**S1–S4 Data**). To attempt to correct this inability to call peaks on the male X chromosome, we adjusted several MACS2 model parameters and settings (see Materials and Methods), and tried additional peak calling algorithms, but were not able to identify many additional peaks on the male X chromosome. We speculate that this may be because of the overall chromatin decondensation and conformational differences of the male X chromosome due to dosage compensation, leading to difficulties in peak calling [56,57].

**Fig 2:**
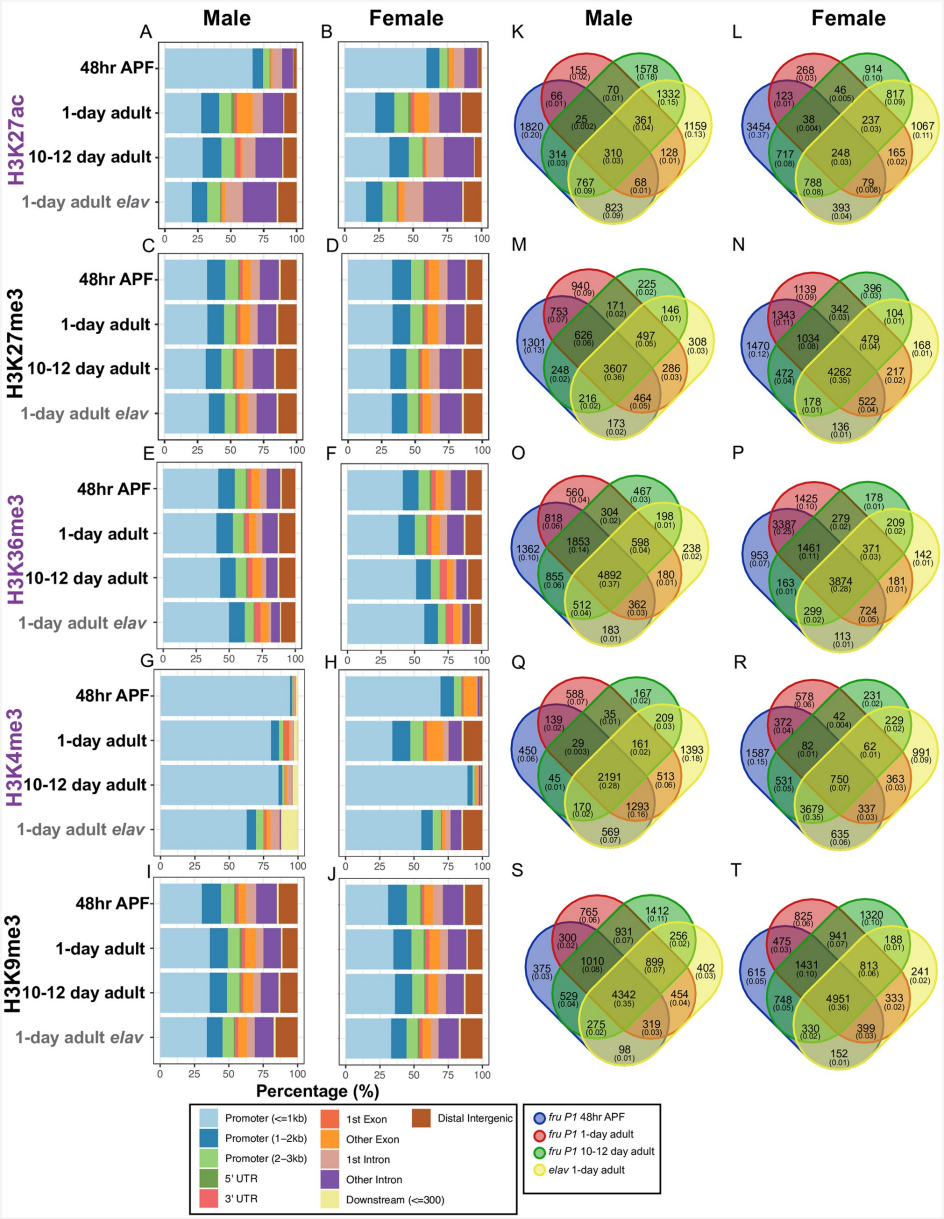
Genomic feature distribution and overlap of genes containing histone H3 chromatin modifications. **(A-J)** Genomic feature distributions of MACS2 peaks for activating (H3K27ac, H3K36me3, H3K4me3; purple) and repressive (H3K27me3 and H3K9me3; black) H3 chromatin modifications. The histone modification is indicated on the left. Each panel includes data for *fru P1* (black) and *elav* neurons (gray) in males (left) and females (right). The X-axis shows the percent of each genomic feature defined in the legend (bottom). **(K-T)** The histone modification is indicated on the left. Venn diagrams showing overlap of genes containing MACS2 peaks across time points in *fru P1* and *elav* neurons. The male (left) and female (right) data are indicated at top. The legend for the Venn diagrams is shown at bottom. For each Venn diagram category, the number of genes, and the proportion of the total genes (in parentheses) in each panel is shown. All MACS2 peaks were called on pooled biological replicates (n = 3–4) and identified as enriched relative to matched input ChIP-seq libraries for each time point, sex, and neuron type. All MACS2 peaks in this analysis are listed in **S1–S8 Data**. Venn diagram gene list matrices are listed in **S2 Table**. Abbreviations: 5’ untranslated region (5’ UTR) and 3’ untranslated region (3’ UTR).

We determined where peaks occur, with respect to eleven gene features, and how the distribution of peaks changes proportionally over time (**Fig 2A–2J**). We generally find that the proportion of peaks in the gene features are highly similar between males and females at each time point, for each histone modification, even though we cannot call many peaks on the male X chromosome. This is true for the ChIP-seq data from both the *fru P1* and *elav* data sets. Given the limitation of calling peaks on the male X chromosome, we next compared gene feature peak position within sex. In *fru P1* neurons, H3K27ac and H3K4me3 peaks show the largest differences across time points (**Fig 2A–2J**). For example, the male and female data from 48hr APF have ~65% of H3K27ac peaks less than one kb from promotor regions, whereas in adult stages ~34% are less than one kb from the promoter, with a larger percentage of peaks now found in exons, introns, and intergenic regions (**Fig 2A and 2B**). Similarly, for H3K4me3 at 48hr APF in *fru P1* neurons, the majority of H3K4me3 peaks in both males (~95%) and females (~70%) are one kb from promotor regions. Then, the percentage of H3K4me3 peaks with positions distant from the promotor increases in 1-day adults and subsequently decreases at 10–12 days, with the changes more pronounced in females (**Fig 2G and 2H**). For H3K27me3, H3K36me3, and H3K9me3 we observe smaller changes in the proportion of peaks for the gene features across developmental time (**Fig 2C–2F, 2I and 2J**). When we performed this same analysis on the consensus peaks, we obtained nearly identical gene feature distribution results (**S4 Fig**).

Given that some modifications have substantial changes in the distribution of peaks over developmental time in *fru P1* neurons (H3K27ac and H3K4me3), we next determined if this is due to chromatin modifications in different genes or in the same genes, including both the *fru P1* and *elav* data sets in the analysis (**Fig 2K–2T** and **S2 Table)**. The observation that peaks are in different genes for the 1-day time point for the *fru P1* and *elav* chromatin data sets shows that there are cell-type-specific genes with each histone modification. We also generated Venn diagrams to examine the overlap in *fru P1* neurons only (**S5A–S5J Fig** and **S2 Table**). Across the three time points, the genes with H3K27ac peaks have low overlap in males (4% overlap) and in females (3% overlap). The genes with H3K4me3 peaks have more overlap across the three time points in males (34% overlap), than females (9% overlap), which may be related to the observation that the distribution of the peaks have fewer changes in males over time (**S5A–S5J Fig**). For the modifications where the distribution of gene feature distribution does not change substantially over the three time points (H3K27me3, H3K36me3, and H3K9me3), there is also more overlap across the three time points for genes with these modifications (range is 40–51% overlap; **S5A–S5J Fig**). The H3K27ac and H3K4me3 activating modifications are largely found in different genes across time points and thus are not likely to be mediating cell fate determination, whereas the H3K27me3, H3K36me3, and H3K9me3 modifications persist across three time points, suggesting these chromatin modifications may be part of a mechanism to direct cell fate determination.

To determine the roles of genes with modifications that persist across all three time points in *fru P1* neurons (ten gene lists from center of Venn diagrams in **S5A–S5J Fig**), we performed Gene Ontology (GO) enrichment analysis (**S3 Table**). Notably, we find GO enrichment for genes involved in “cell fate commitment” for all gene lists (**S6 Fig** and **S3 Table**). When we examined the top ten GO enrichment terms across the ten lists, we find that many overlap (**S6 Fig**). For example, terms such as “DNA-binding transcription factor activity” and several neuron development terms like “neuron projection development”, “axon development”, and “axon guidance” are enriched across all ten gene lists (**S6 Fig**). The observation that the top GO enrichment terms are similar for the lists of genes that have either persistent activating or repressive modifications suggest that both types of regulation are important for maintaining cell fate.

### Bivalent promoter regions occur near developmental genes in *fru P1* neurons

Next, we examined genes with bivalent promoter regions, defined here as genes that had both an activating H3K4me3 and repressive H3K27me3 histone modification peak within 3kb of the promoter (**S1 Table**). The co-occurrence of these modifications has been suggested to create a poised gene expression state and are often found near developmentally important genes [58,59]. We examine the data from *fru P1* neurons and find that the highest number of genes with bivalent promotors are found in 48hr APF samples (1229 genes in males and 3434 genes in females), with fewer at later time points (1-day adult: 1207 in males, 1208 in females; 10–12 day adult: 413 in males, 1080 in females). There is also little overlap of genes with bivalent promotor regions that persist across all time points (males: 89 genes, females: 100 genes) (**S5K Fig**). When we performed GO enrichment analysis on the lists of genes with bivalent promoter regions from each time point, we find that “cell differentiation” and terms related to neuron developmental processes are among the top ten GO terms for all lists (**S3 Table**).

Additionally, we determined if the lists of genes with bivalent promoters are enriched with those that encode for products with different protein domains. We find that protein domain enrichment differs between the earlier time points and 10–12 day adults in both sexes. For 48hr APF and 1-day adults there is enrichment for “homeobox” and “zinc finger” domain encoding genes, whereas at 10–12 days we see the highest enrichment for genes that encode products in the “immunoglobulin-like domain superfamily” (**S3 Table**). Genes in this family encode cell surface proteins that are involved in cell adhesion [60,61]. This is consistent with our recent study that showed an interacting subfamily of immunoglobulin-fold domain encoding genes, *dpr*s and *DIP*s, are expressed in adult *fru P1* neurons [62]. The observation that immunoglobulin-like domain superfamily encoding genes are in a poised gene expression state may suggest a role in mediating neuronal plasticity in adult *fru P1* neurons.

### Hierarchical clustering of genome-wide chromatin modifications in *fru P1* and *elav* neurons reveal stage- and sex-dependent differences

**Fig 3:**
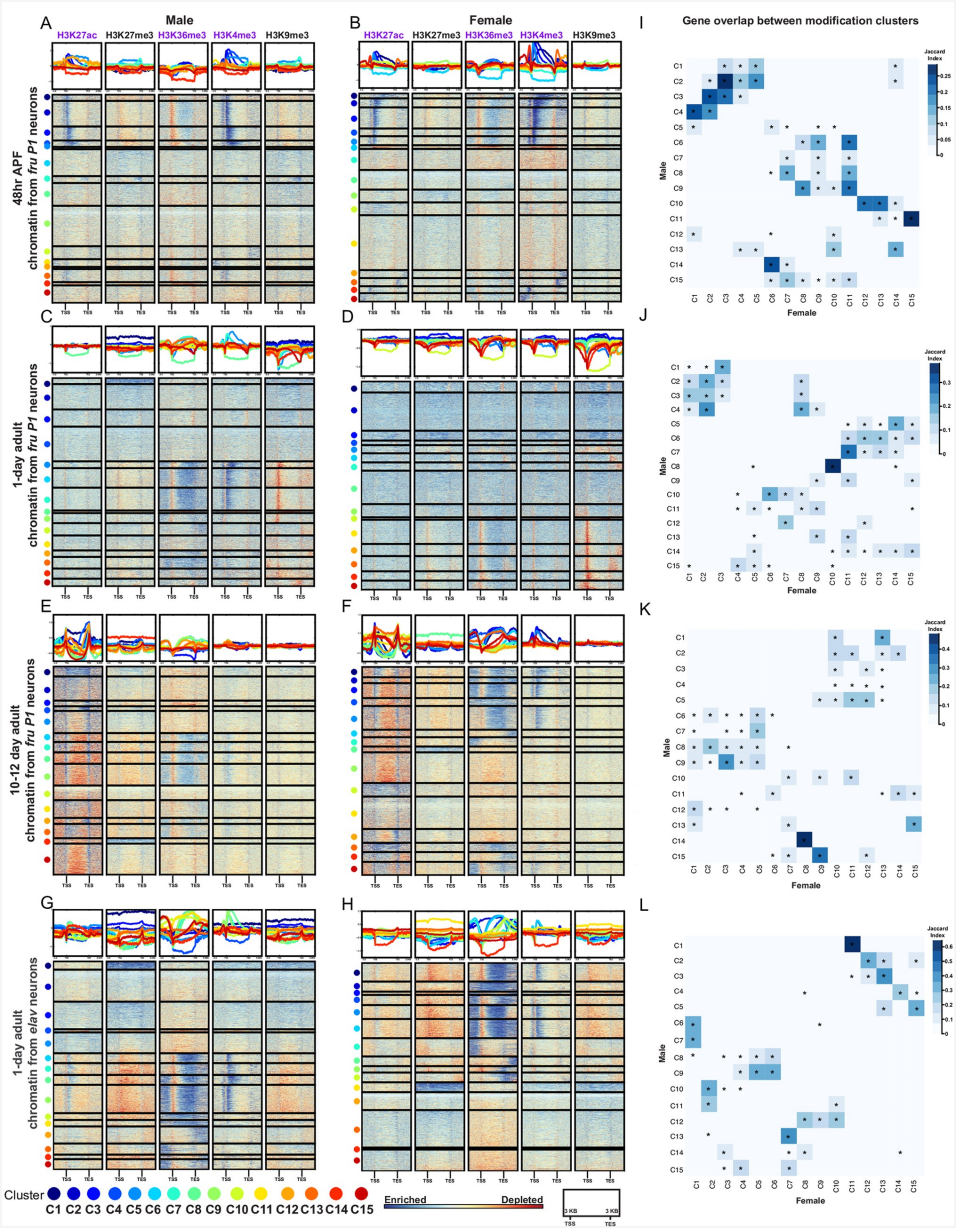
Genome-wide hierarchical clustering of histone modification distributions and cluster overlaps between the sexes. **(A-F)** Hierarchal clustering reveals shared combinations of histone modifications on all annotated genes, generated using deepTools. The ChIP enrichment of the input normalized data is indicated on the color bar, from blue (enrichment) to red (depletion), calculated in 50bp bins. The genes (rows) are clustered by the similarity of the ChIP patterns across all five histone modifications (each cluster number marked with a colored dot). The heatmap is divided into five columns, one for each chromatin modification. The top panel shows the average distribution of a chromatin modification in each cluster, with the line color indicating the cluster number. For each chromatin modification, the heatmap shows scaled gene bodies (scaled to 5kb) and 3kb upstream and downstream of the transcriptional start site (TSS) and transcriptional end site (TES). Heatmaps for *fru P1* chromatin data from 48hr APF **(A-B)**, 1-day **(C-D)**, and 10–12 day **(E-F)**. Heatmaps for *elav* chromatin data from 1-day **(G-H)**. The sex is indicated at the top. **(I-L)** GeneOverlap heatmap [66], where color indicates the Jaccard Index score, showing the similarity of the gene lists between clusters in males and females. For each panel the X-axis indicates female cluster number and the Y-axis indicates the male cluster number (color legend for clusters on the bottom). The legend for the Jaccard similarity index is on the right of each panel. Asterisks indicate statistically significant overlap of genes between clusters (Fisher’s exact test, p<0.05). Gene list information for all heatmap clusters is available in **S4 Table**. Gene overlap index and statistics are available in **S5 Table**.

Next, using hierarchical clustering we examined the combinations of histone modifications on all annotated genes (**Fig 3** and **S4 Table**). The heatmaps show clustered genes that are grouped based on similarity across the five chromatin modifications. This was performed using the input normalized data generated using deepTools, which calculates enriched and depleted ChIP regions over 50bp bins, relative to input, and is effective genome wide. The heatmaps show the ChIP enrichment and depletion, 3kb upstream of the start of transcription (TSS), the length of the normalized gene body (shown as 5kb) and 3kb downstream of the transcriptional end (TES).

First, these heatmaps provide further validation of the approach, given that overall the positions where there are enrichments of ChIP signal are consistent with the known locations for these modifications in *Drosophila* [53,63]. For the activating modifications, we find H3K27ac near the TSS, H3K4me3 near the TSS, and H3K36me3 along the gene body. Both repressive modifications show less localized enrichment patterns, which may be because we are examining genes, and not pericentric and repetitive regions which are typically heterochromatic [64]. We do find that H3K9me3 has pronounced depletion at the TSS in some genes at the 1-day time point, which is unique compared to other stages examined here (**Fig 3**). One unexpected finding is the presence of H3K27ac peaks at the TES in 10–12 day adults, which to our knowledge is not widely reported.

Overall, the enrichment patterns of activating chromatin modifications in *fru P1* neurons show the largest differences across time points and not between the sexes, which is not unexpected given that most *Drosophila* genes do not have sex-specific expression patterns in *fru P1* neurons (see TRAP section below). For example, in both sexes at 48hr APF, H3K27ac enrichment mainly surrounds the TSS, in the sets of clusters with most pronounced ChIP-seq enrichment (**Fig 3A and 3B**, dark blue). At 1-day, the H3K27ac enrichment is more diffuse in pattern (**Fig 3C and 3D**). By the 10–12 day time point, H3K27ac is enriched near both the TSS and TES (dark blue), and this is observed for the majority of genes (**Fig 3E and 3F**). In addition, in both sexes at 48hr APF, the genes with the most pronounced H3K27ac enrichment near the TSS (dark blue) also have the most pronounced H3K4me3 enrichment near the TSS and along the gene body (dark blue) (**Fig 3A and 3B**). Furthermore, H3K36me3 shows changes over developmental time, with high enrichment along the entire gene body seen at the later two time points and is not as pronounced at the 48hr APF time point (**Fig 3A–3F**).

The repressive modifications also show time point specific differences. Notably, there is a set of genes with H3K27me3 modification enrichment along the length of the gene found only in the adult data sets (**Fig 3C–3H; 3C**-C1, **3D**-C3, **3E**-C14, **3F**2 C8, **3G**-C1, and **3H-**C11). Interestingly, GO enrichment analysis to determine if there are overrepresented genes with known functions reveals that the list is enriched with “homeobox domain” encoding genes [65], which suggests that these critical transcriptional regulators are silenced after pupal stages in neurons, consistent with previous findings [63] (**S3** and **S4 Tables**). Additionally, there are pronounced changes in the pattern of H3K9me3 over developmental time, with the 1-day time point having the most defined patterns of enrichment and depletion over the gene regions examined (**Fig 3C and 3D**).

We also observe minor sex- and cell-type-specific differences in chromatin modifications, at the adult stages. At the 1-day time point, there is H3K4me3 enrichment at the TSS in males that is not as pronounced as in females (**Fig 3C and 3D**). Then at the 10–12 day time point, there is more pronounced H3K4me3 enrichment near the TSS in females (**Fig 3E and 3F**). The sex differences described are not observed in the *elav* neuron data, consistent with *fru P1* neurons having sex-specific identity (**Fig 3C, 3D, 3G and 3H**). The overall pattern between *fru P1* neurons and *elav* neurons at the 1-day time point are different, with all the modifications in the *elav* data showing more pronounced enrichment and depletion along the gene regions examined, for many genes (**Fig 3C, 3D, 3G and 3H**).

We next determined if the male and female clusters with similar patterns of histone modifications contain the same genes. To do this, for each time point we examined the overlap of genes between clusters from both sexes, using the GeneOverlap analysis tool (**Fig 3I–3L** and **S5 Table**) [66]. The blue color scale indicates the Jaccard similarity coefficient, and the asterisk indicates the significance of the overlap based on a Fisher’s exact test (p<0.05). The clusters in the heatmap with the most overlapping genes between the sexes tend to have similar patterns of histone modification enrichment (**Fig 3I–3L**, darkest blue boxes). For example, at 48hr APF, clusters 1–4 in males, and clusters 1–5 in females have similar H3K27ac, H3K36me3 and H3K4me3 patterns and several of these clusters have significant overlap in genes (**Fig 3A, 3B** and **3I**). We also find clusters that have significant overlap between males and females but have different patterns of histone modifications (**Fig 3I–3L**). One example of this is at 48hr APF, where there is a significant overlap of genes between male cluster 11 and female clusters 13 and 14, but different patterns of enrichment for H3K27ac and H3K4me3 (**Fig 3A, 3B** and **3I**). However, male cluster 11 also has significant gene overlap with female cluster 15 and the same patterns of histone modifications. *fru P1* chromatin data sets have more clusters with significant gene overlap between the sexes than the *elav* chromatin data sets (**Fig 3I–3L**). In all adult *fru P1* and *elav* data sets, the clusters with repressive modifications containing the homeobox genes have a highly similar set of genes between males and females (**Fig 3C–3H and 3J–3L**). Overall, we observe that the majority of clusters with a similar modification enrichment pattern in both sexes share a significant number of genes. There is a smaller subset of clusters with significant gene overlap that have different histone modification profiles.

### TRAP RNA-seq identifies mRNAs that are enriched in *fru P1* neurons in a stage-specific manner

Next, we examined gene expression specifically in *fru P1* neurons from males and females, using the translating ribosome affinity purification (TRAP) approach [41]. The TRAP approach allows one to enrich for mRNAs that are actively translated in a cell-type-specific manner, via Gal4/UAS expression of a GFP-tagged ribosomal subunit (RpL10a). We examined all *fru P1* neurons from the 48hr APF stage and *fru P1* neurons from adult heads at the 10–12 adult stage, which is equivalent to the *fru P1* neurons profiled in the chromatin data sets. We have previously described *fru P1* TRAP gene expression results for neurons from 1-day adult heads and present those results in comparisons here [42]. For each time point, we performed gene expression analysis at the exon-level, to account for transcript-isoform expression differences. A gene was considered differentially enriched if at least one exon had differential expression (FDR <0.2).

At each time point, to identify genes with enriched expression in *fru P1* neurons, in either males or females, we performed two statistical comparisons: 1) male TRAP vs. male total mRNA; and 2) female TRAP vs. female total mRNA, which is similar to the comparisons for the chromatin data sets (hereafter called TRAP-enriched; **S6–S8 Tables**). We also present sex differences in either TRAP or total mRNA: 1) male TRAP vs. female TRAP (hereafter TRAP-biased); and 2) male total mRNA vs. female total mRNA (**S9 and S10 Tables**).

As expected, some *fru* exons, including the *fru P1*-specific exons, are enriched in the TRAP samples, relative to total mRNA control (**S6 and S7 Tables**). In 48hr APF TRAP vs. total mRNA, the *fru P1* specific exons are enriched in TRAP samples, but do not meet the threshold of significance, whereas other *fru* exons show significant enrichment (**S6 Table**). In 10-day TRAP vs. total mRNA, *fru P1* and other *fru* exons are significantly enriched in TRAP samples, providing overall validation of the approach (**S7 Table**). When we examine the mapped reads at the *fru* locus at 48hr APF and 10–12 days, the sex-specifically spliced gene region shows the expected abundance of reads, with more in females, since the retained exonic region is larger in females (**S7 Fig**), similar to our observations at 1-day [42]. Additionally, as further confirmation of the approach, the male and female TRAP-enriched and TRAP-biased gene lists significantly overlap with lists of genes that are predicted to be direct Fru^M^ targets (**S5 Table**) [67,68]. Moreover, we find expression of *engrailed* in our TRAP-enriched data which has been shown to be expressed in a subset of Fru^M^-expressing neurons in a single-gene study [69]. Here, an analysis of the genes that are more highly expressed in *fru P1* neurons vs. total mRNA (TRAP-enriched) and TRAP male and female-biased genes (TRAP-biased) is presented. The other gene lists from additional comparisons are provided (**S6–S10 Tables**).

**Fig 4:**
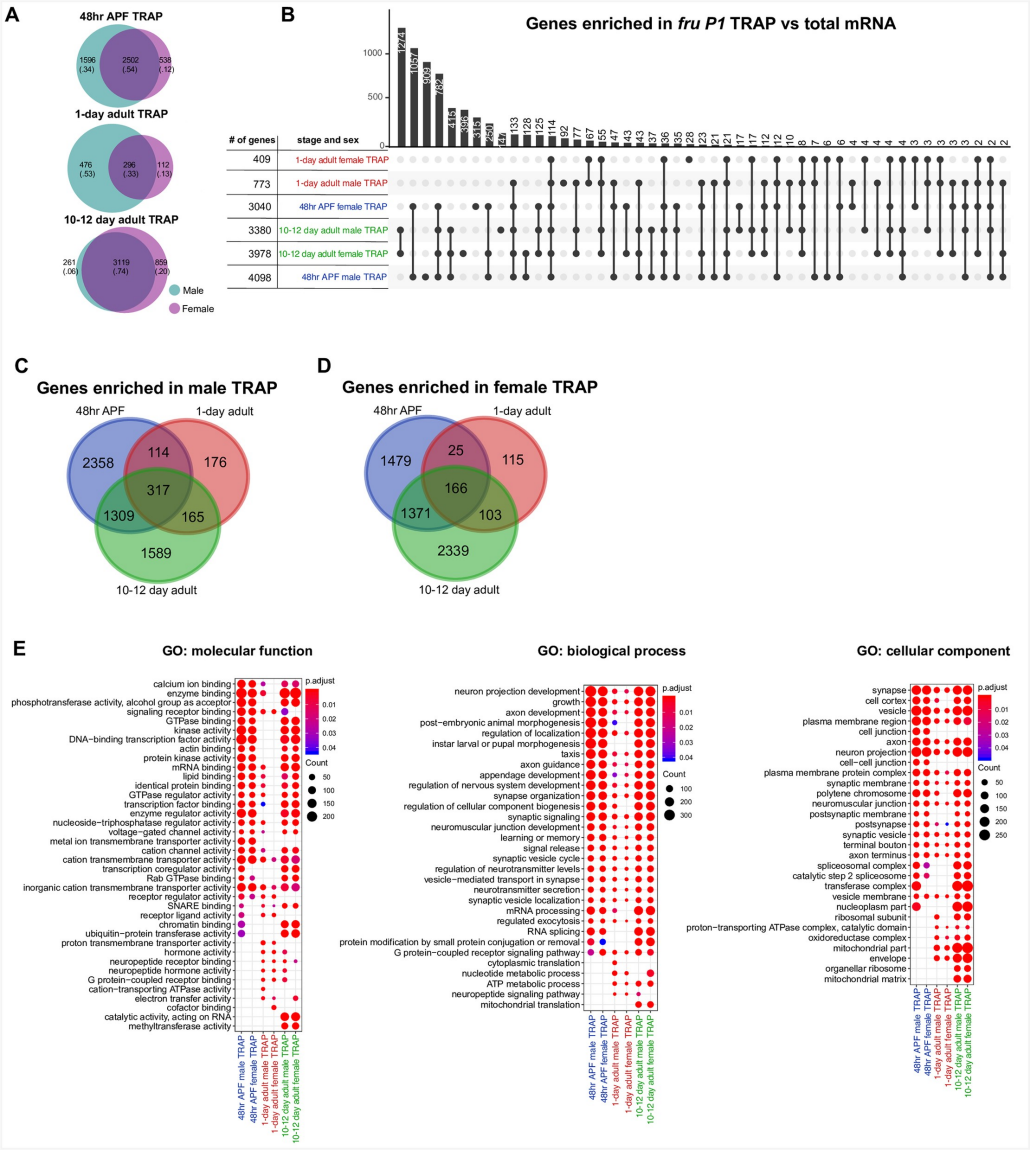
TRAP RNA-seq identifies mRNAs that are enriched in *fru P1* neurons at multiple time points. Analysis of TRAP-enriched genes. These genes were identified based on having at least one exon with significantly higher expression in TRAP vs. total mRNA (FDR<0.2). **(A)** Venn diagrams comparing TRAP-enriched genes between males (teal) and females (purple) at three time points. For each Venn diagram category, the number of genes, and the proportion of the total genes (in parentheses) in each panel is shown. **(B)** Upset intersection plot of TRAP-enriched genes from 48hr APF (blue), 1-day (red) and 10–12 day (green) time points. n = 4–5 biological replicates per condition. (**C-D**) Venn diagrams comparing TRAP-enriched genes across time points for each sex. (**E**) Gene Ontology (GO) enrichment analysis for TRAP-enriched genes. The GO categories are molecular function, biological process, and cellular component. The GO terms shown in the plots are the top ten most significantly enriched term for each list (non-redundant shown; Benjamini-Hochberg, p<0.05). The size of each dot indicates the number of genes (count) and the color indicates the p value (p.adjust). All GO information is in **S3 Table**. Gene lists are in **S6 and S7 Tables**.

First, we compared the number of genes with enriched expression in *fru P1* neurons that are male TRAP-enriched, female TRAP-enriched, or TRAP-enriched in both sexes, using Venn diagrams (**Fig 4A** and **S6 and S7 Tables**). Given that each time point has a different overall number of genes with TRAP-enriched expression, we also report the proportions of genes with enriched expression in each category to facilitate comparisons across the time points. We find a higher percent of enriched genes in male TRAP-enriched lists than female TRAP-enriched lists at 48hr APF and in 1-day adults (**Fig 4A**). At 48hr APF, 34% of genes are male TRAP-enriched, 12% are female TRAP-enriched and 54% are enriched in both male and female TRAP samples. At 1-day, 53% of genes are male-enriched, 13% are female-enriched and 33% are enriched in both male and female TRAP samples. In contrast, at the 10-12-day time point, a large fraction of genes have TRAP-enriched expression in both sexes (74%) and fewer male TRAP-enriched (6%) than female TRAP-enriched (20%). While there is a shared set of genes between males and females with *fru P1* TRAP-enriched expression at each stage, 48hr APF and 1-day adults show a higher fraction of genes with male TRAP-enriched expression. This is consistent with our previous observations where we suggest that this could be due to Fru^M^ gene regulation in males [42]. This result is also in line with previous work showing *fru P1* has a critical function during development for reproductive behaviors [28,29,70].

To determine if there is overlap between genes that have *fru P1* TRAP-enriched expression in the gene lists from the different stages and sexes, we generated an UpSet plot [71], which is conceptually similar to a Venn diagram, but can accommodate a large number of conditions (**Fig 4B**). The histogram at the top of the UpSet plot shows the number of genes with overlap in the gene sets (**Fig 4B**). The dots beneath each bar show the gene sets that have the overlapping genes (**Fig 4B**). For example, the 10-12-day male and female *fru P1* TRAP-enriched data sets have a large number of genes that overlap (1,274 genes), as expected given the overlap shown in the Venn diagram (3,119 genes, **Fig 4A**). A similar result is shown for the 48hr APF TRAP-enriched data, with a large number of genes overlapping in the male and female *fru P1* TRAP-enriched genes (1,057 genes). The observation of sex-shared TRAP-enriched genes suggests that expression of stage-specific genes is important, in both males and females.

We conducted GO term analysis on the *fru P1* TRAP-enriched genes to determine if there were genes with known functions overrepresented in the lists, examining the top ten terms for each list (**Fig 4E** and **S3 Table**). We examined GO term categories: ‘molecular function’, ‘biological process’ and ‘cellular component’. Given there is overlap across all the gene lists, many of the GO terms are shared and include several expected of neurons, though there are some unique terms for each time point (**Fig 4E**). For ‘molecular function’, the lists from the 1-day time points have several unique terms including ‘neuropeptide receptor binding’ and ‘hormone activity’ terms, and fewer shared terms. The 10–12 day male and female, and 48hr male *fru P1*-enriched lists have chromatin binding as significant. For ‘biological process’ and ‘cellular component’, the lists from the adult samples, have ‘metabolic process’ terms and ‘mitochondrial’ terms that are unique. We also note that the 48hr APF and 10–12 day TRAP-enriched gene sets are enriched with genes that encode Immunoglobulin domain containing proteins/cell adhesion molecules (IgSFs), as we previously reported for 1-day adults [42]. We have also validated that the Dpr/DIP IgSFs, a subset of these cell adhesion molecules, play a role in *fru P1* neurons, further validating the data sets presented here [62].

We next examined the genes with sex-biased expression in TRAP samples, which were identified by comparing expression between the male and female TRAP samples. Given that the analysis was done on the exon level, we can find genes for which one exon may be male-biased and another female-biased, so the gene would be considered to have a transcript isoform with sex-biased expression in each sex (**S9 and S10 Tables**). For example, at the 48hr APF stage there are 43 genes that have transcript isoforms with sex-biased expression in each sex, including *Sxl*, which is known to have male- and female-specific transcript isoforms [72]. Additionally, we find a larger number of male-biased genes at 48hr APF (~1.4 fold), which is similar to what we reported for 1-day (~4.0 fold) [42], but at 10–12 there are fewer genes with sex-biased expression and more in females (~1.3 fold) (**S7C Fig**). Further, more genes overlap between the 48hr APF and 1-day time points, in males and females, which might be expected given the small number of genes with sex-biased expression at 10–12 day (**S7D–S7F Fig**). We conducted GO term analysis on the *fru P1* TRAP-biased genes to determine if there were genes with known functions overrepresented in the lists and show the top ten GO terms for each list (**S7E Fig** and **S3 Table**). We find that the 10–12 day TRAP-biased gene lists have more unique GO terms, with several categories of genes expected to underlie adult behavior. Overall, this analysis demonstrates the importance of performing stage-specific analyses, given the differences in genes observed at each time point.

### Hierarchical clustering analysis of chromatin modifications for genes with mRNAs that are enriched in TRAP gene expression data sets

**Fig 5:**
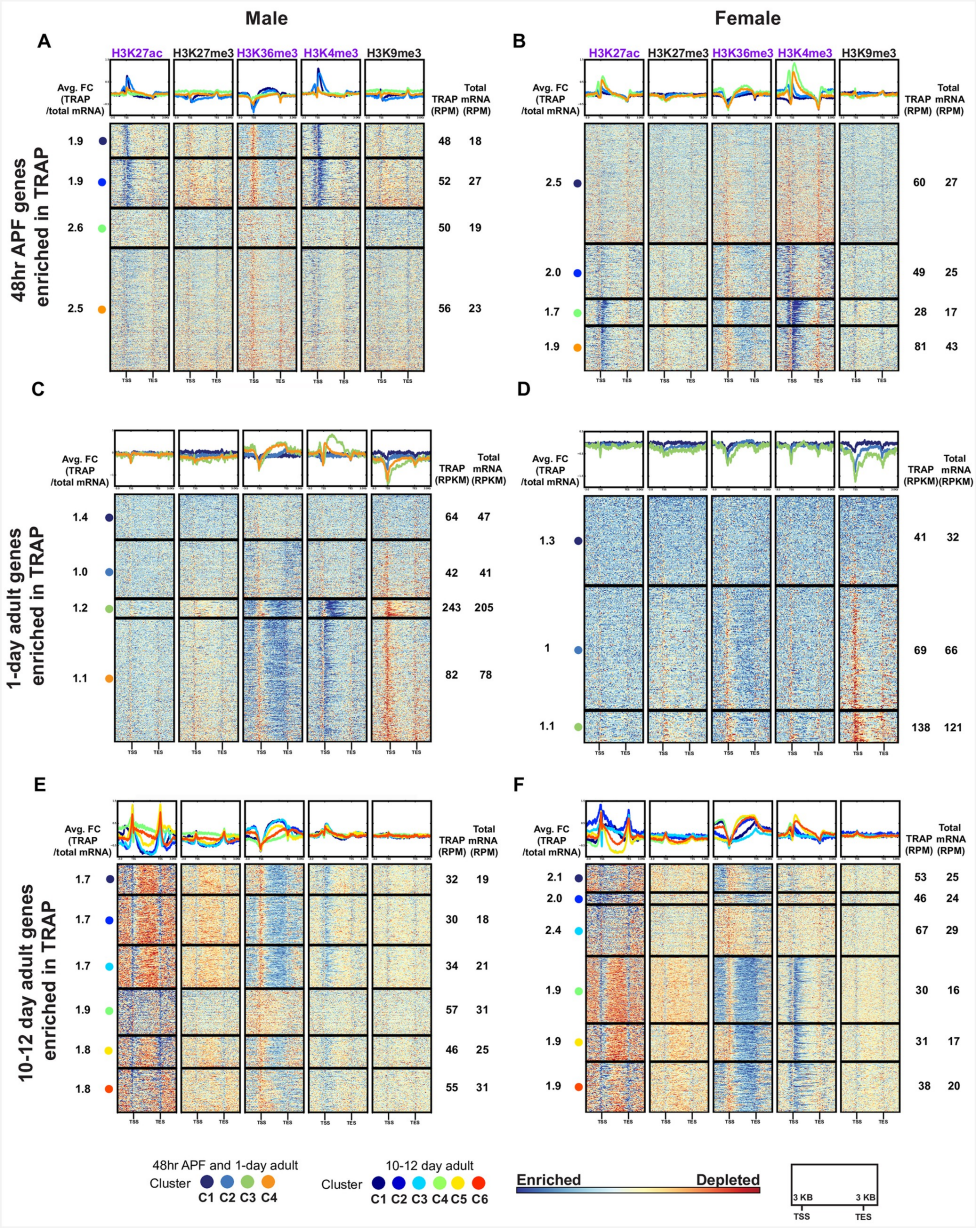
Hierarchical clustering of histone modification distributions for TRAP-enriched genes. Hierarchal clustering reveals shared combinations of histone modifications for TRAP-enriched genes, generated using deepTools. For detailed description of heatmaps see **Fig 3**. Heatmaps for *fru P1* chromatin data from 48hr APF **(A-B)**, 1-day **(C-D)**, and 10–12 day **(E-F)**. Gene lists for each cluster are provided in **S4 Table**. For each cluster of genes, the average fold-enrichment (Avg FC) of gene expression is indicated on the left (TRAP-enrichment), calculated at the exon level. For each cluster of genes, the average expression is indicated on the right for the TRAP and total mRNA data, calculated at the exon level. The 48hr APF and 10–12 day gene expression levels are provided as reads per million (RPM). The 1-day timepoint gene expression levels are provided as reads per kilobase per million (RPKM) [42]. For TRAP expression data see **S6 and S7 Tables**.

We next determined the pattern of chromatin modifications for genes with *fru P1* and *elav* TRAP-enriched expression at each time point. We examined heatmaps to see the patterns of chromatin modifications, generated with the deepTools algorithm (**Fig 5**), as above. The patterns of chromatin modifications are similar for the male TRAP- and female TRAP-enriched genes, within time point (**Fig 5A–5F**), as was true when all genes were examined (**Fig 3**). Furthermore, within each time point, there is no pattern of histone modifications that would clearly predict higher fold-enriched expression of a gene in *fru P1* neurons, with average fold differences in expression indicated on the left of each heatmap (**Fig 5A–5F**). However, unexpectedly, at the 48hr time point, genes in a cluster with average 2-fold or greater *fru P1* TRAP-enriched expression have less enrichment of activating chromatin modifications H3K27ac and H3K4me3 (**Fig 5A and 5B**). We observe similar results with the *elav* chromatin data, where *elav* TRAP-enriched genes have similar histone modification patterns (**S8 Fig**) [41], but no large differences in the patterns that would predict gene expression levels.

It appears that the chromatin modification patterns for TRAP-enriched genes indicate gene expression, but not levels of expression. To further address this, we examined the chromatin modification patterns for the genes with higher expression in total mRNA than TRAP-enriched mRNA. These genes have the same patterns of chromatin modifications as the TRAP-enriched genes (**S8 and S9 Figs**), for both *fru P1* and *elav* data sets. Overall, we find histone modification patterns associated with gene expression, but no patterns appear to be predictive of different levels of expression in *fru P1* or *elav* neurons. This result is also further confirmed by heatmaps made with genes that have detected expression in *fru P1* or *elav* neurons, rather than TRAP-enriched or total mRNA-enriched (**S10 Fig)**. This demonstrates that the histone modifications patterns may be predictive of gene expression in *fru P1* and *elav* neurons but are not predictive of gene expression levels.

One goal of this study was to examine how Fru^M^ impacts chromatin modifications, given a previous study showed that Fru^M^ recruits chromatin modifying enzymes [33]. To assess if genes that are predicted to be directly bound by Fru^M^ have different chromatin modification enrichment profiles, we determined which clusters of *fru P1* TRAP-enriched genes have significant overlap with lists of genes that are predicted to be direct Fru^M^ targets [67,68] (**S5 Table**). Based on visual inspection, the clusters with the more robust enrichment of activating chromatin modifications tend to be those that contain genes that are less likely to be direct Fru^M^ targets. Given that this is observed in heatmaps from both males and females, this suggests the chromatin modification pattern differences in these clusters are not simply due to Fru^M^ binding activity.

### Identification of H3K27ac super-enhancers in chromatin from *fru P1* and *elav* neurons

**Fig 6:**
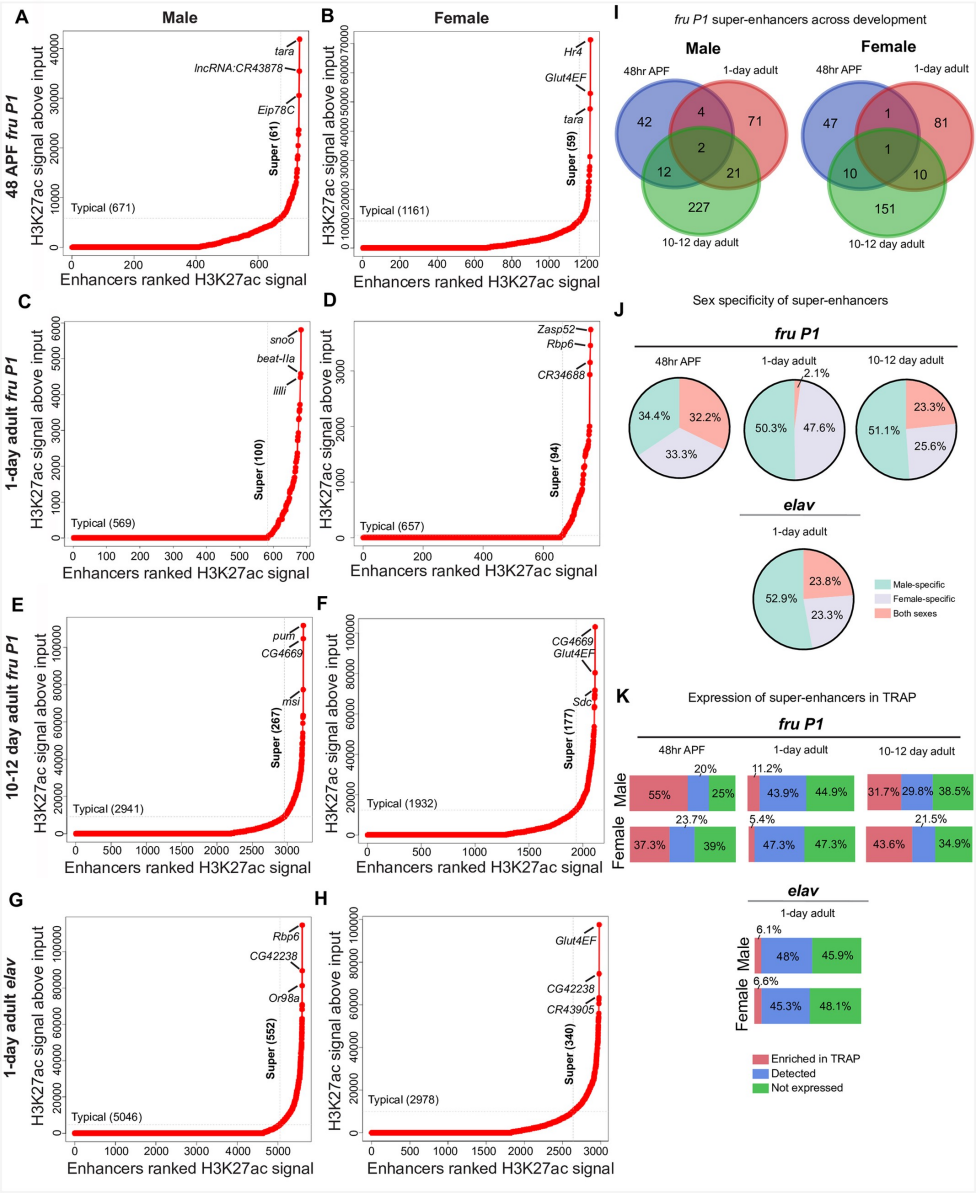
Super-enhancers identified based on H3K27ac peaks in *fru P1*- and *elav* neuron chromatin data sets. Super-enhancers (SEs) in *fru P1* **(A-F)** and *elav* (**G-H**) neuron chromatin data, identified using H3K27ac MACS2 peaks and the ROSE algorithm [73,74]. The ROSE algorithm was implemented with 12.5kb regions stitched together and exclusion of regions +/- 2500bp to the TSS. The X-axis has the enhancers ranked by H3K27ac signal. The Y-axis indicates the H3K27ac signal above input. Dotted lines indicate the boundary between typical enhancers and SEs. The top 3 ranking SEs for each condition are indicated. **(I)** Venn diagrams showing the overlap between SE-containing genes across time points for *fru P1* chromatin data from males (left) and females (right). **(J)** Percentages of SE-containing genes for each stage and neuron type (*fru P1* or *elav*) that are male-specific, female-specific or occur in both sexes. **(K)** Percentages of SE-containing genes with detected or TRAP-enriched gene expression in *fru P1* or *elav* expression data sets.

Super-enhancers (SEs) are defined as regions of clustered enhancers and have been shown to regulate cell-type-specific gene expression [73]. We identified SEs based on presence of clusters of H3K27ac peaks using the ROSE algorithm [73,74]. In brief, H3K27ac peaks located within a 12.5kb distance from each other were stitched together and plotted in an increasing order, based on their input-normalized H3K27ac ChIP-seq signal. Enhancers above the inflection point of the curve were defined as SEs (**Fig 6A–6H** and **S11 Table**). Given the difficulty calling peaks on the male X chromosome using MACS2, we perform the analysis on all chromosomes (**Fig 6**), and only autosomes (**S11 Fig**), and find overall the trends are similar, regardless of the X chromosome SEs being included. For *fru P1* data sets, we find an increasing number of genes with SEs across developmental time in both males (61–267 SEs) and females (59–177 SEs), with the largest number identified in 10–12 day adult males (**Fig 6A–6F**). We also identified genes with SEs in *elav* chromatin for males (552 SEs) and females (340 SEs) (**Fig 6G and 6H**).

When we compare SEs across time points, we find that the majority of the genes with SEs are stage-specific, as shown in the Venn diagrams (**Fig 6I**). Furthermore, genes with SEs also significantly overlap with lists of genes that are predicted to be direct Fru^M^ targets (**S5 Table**) [67,68]. The GO analysis of genes with SEs at 48hr APF reveals several enriched ‘neuron developmental’ terms, which are also enriched at 10–12 days. However, at 10–12 days we find additional enriched ‘adult behavior’ and ‘signaling’ terms (**S3 Table**). We next determined if there is sex-specificity of genes with SEs at each time point (**Fig 6J)**. Males and females have a similar number of sex-specific genes with SEs at the early time points, but the 10–12 day time point has more genes with SEs that are unique to males (**Fig 6J)**. For the *elav* data sets, genes with SEs also have more male unique genes (**Fig 6J)**. However, we find that having a SE is not highly predictive of gene expression, when we compare to the *fru P1* and *elav* TRAP data sets from each time point (**Fig 6K**). Across the time points, we find a range of SE-containing genes that also have significantly enriched expression in the *fru P1* TRAP-enriched data sets (5.4%-55%; red squares in **Fig 6K**). This also true for genes with SEs in the *elav* data set, with only 6% of genes having enriched expression in the *elav* TRAP-enriched data set [41]. Taken together, a gene containing a SE does not predict expression in the neurons at the time points examined.

### Candidate genes with super-enhancers and TRAP-enriched expression are expressed in *fru P1* neurons

**Fig 7:**
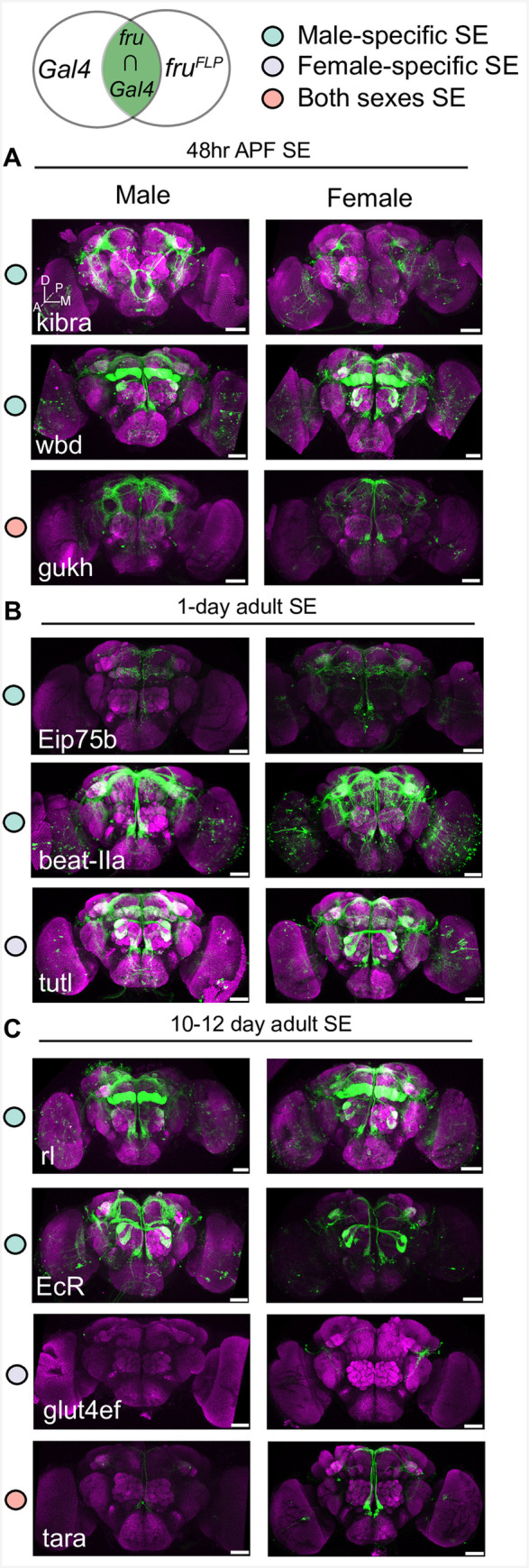
Co-expression of super-enhancer containing genes and *fru P1* in neurons. Super-enhancer containing genes were identified at each time point and their co-expression with *fru P1* was determined in 10–12 day adult brains. The top illustration shows the genetic intersectional approach where membrane bound GFP is only expressed in neurons that co-express the candidate gene and *fru P1*. The top of each set of panels indicates the time point for which the super-enhancer was detected in the chromatin data sets: **(A)** 48hr APF stage, **(B)** 1-day adult, and **(C)** 10–12 day adult stage. The gene name is indicated in each panel, with intersecting expression patterns shown for male (left) and female (right) brains. Colored dots in legend indicate that the SE was identified in chromatin data from either male, female or both sexes. Three-dimensional confocal maximum projections from successive confocal slices are presented with the most anterior slice positioned first. Brain anatomical axes are presented on lower left of first brain image panel (see **A**): D, *dorsal*; M, *medial*; A, *anterior*; P, *posterior*. Further information on brain position and structure has been previously described [90]. Scale bars = 50 μm.

Some genes were identified as having SEs, based on MACS2 H3K27ac peaks, and also having TRAP-enriched expression, so are a high confidence set of genes to determine if they overlap with *fru P1* expression. To determine co-expression, we used an intersectional genetic strategy, which is a modification of the Gal4/UAS system, with expression of a membrane-bound GFP marker indicating co-expression. The UAS-GFP reporter transgene has a stop cassette that can be removed by *fru P1* driven *flippase* expression [75]. Once the stop cassette is removed the reporter gene expression is driven by a candidate-gene-specific-Gal4 driver (**Fig 7A**). We selected genes where there are stocks available that have *Gal4* transgenes [76,77]. We examined 10–12 day adult brains, where we can detect perdurance or concurrent expression of GFP. Nine out of 10 genes we screened exhibit expression in both sexes, consistent with our TRAP-enriched expression results (**S6 and S7 Tables**). *glut4ef* only shows expression in female *fru P1* neurons, which might be due to the nearby female-specific SE identified at the 10–12 day stage that is not detected in males. However, *glut4ef* is TRAP-enriched in both males and females. This analysis also reveals sexual dimorphism in the co-expression patterns that could be due to differences in gene expression, but that remains to be determined. For example, seven lines with sex-specific SEs, have clear sexual dimorphisms apparent (*kibra*, *Eip75b*, *beat-IIa*, *rl*, *EcR*, *wbd*, and *glut4ef*). Though it should be noted that genes with SEs in both sexes also show sexual dimorphisms in their expression pattern (*gukh* and *tara*). Overall, all the candidate genes with SEs and TRAP-enriched expression had overlapping *fru P1* expression, demonstrating that these are a high confidence set of genes that are co-expressed with *fru P1*.

## Discussion

Here, we describe Chromatag, a new molecular-genetic tool to perform cell-type-specific chromatin modification analyses that complements other existing approaches [34,35]. A benefit of the Chromatag approach is that FACS purification is not needed, so the tissues are not perturbed before the chromatin fixation step and thus may better reflect the true chromatin state. Furthermore, this tool can be used with any Gal4 driver to examine any cell-type-specific chromatin modification or chromatin binding protein, for which there is a ChIP-grade antibody available, so is highly versatile. The wealth of Gal4 driver lines in *Drosophila* and the additional tools available to further restrict Gal4 expression opens up several possibilities to deepen our understanding of chromatin modifications and chromatin binding proteins. The results shown here demonstrate the approach is effective in small sets of neurons, at several developmental stages.

Our examination of five chromatin modifications in *fru P1* neurons, from three time points, revealed that there are large changes in chromatin modification profiles over developmental time and fewer sex-specific differences (**Fig 3**). These results show the importance of understanding the functional differences of chromatin modifications over developmental time, especially with respect to cell fate determination and adult physiological roles. One goal of this work was to determine if there is a chromatin modification pattern that might be predictive of gene expression in *fru P1* neurons. While the overall patterns of chromatin modifications are different for *fru P1* TRAP-enriched genes, as compared to all genes, there was not a highly predictive pattern for gene expression (**Figs 3** and **5**). This observation is consistent with other studies that have shown that activating histone modifications are not always indicative of gene expression or gene expression levels [78,79]. This could be because the five chromatin modifications chosen are not the critical ones that would be indicative of gene expression in the cell-types we analyzed. Furthermore, given that we examined all *fru P1* neurons, we may miss chromatin modification patterns that are unique to a small set of cells, given the averaging of ChIP-seq signal across all neurons. Additionally, if a chromatin modification is transiently present, it may be difficult to detect. Finally, our TRAP-seq gene expression analyses are cell-type-specific but only includes the actively translated subset of mRNAs. Thus, the chromatin modification patterns may reflect overall gene expression that could be missed using TRAP RNA-seq. Given the advent of single-cell chromatin modification approaches, future studies can potentially resolve these issues.

The cell-type-specific TRAP gene expression studies add to the growing body of work that have identified genes important for *fru P1* function and contain a treasure trove to mine for further single-gene studies [42,67,68,80–82]. The new TRAP-seq data sets presented will aid in our understanding of how the potential for behaviors are established and maintained, by comparing the 48hr APF and 10–12 day adult data sets (**Figs 4** and S7). For example, we find many unique genes with TRAP-enriched expression at each time point, within sex. Furthermore, we find more male TRAP-enriched genes, compared to females, during 48hr APF, consistent with a similar observation at 1-day [42]. At 10–12 days, this large skew of male TRAP-enriched genes is not observed. Additionally, the 48hr APF time point has the largest number of male-biased TRAP genes, suggesting a critical role for Fru^M^ during metamorphosis and early adult stages, which is also consistent with previous functional studies [28,29,70]. Most GO terms identified at 48hr APF for TRAP-enriched genes are also found at 10–12 days, though there are several unique terms at 10–12 days. When we closely examined the GO enrichment for these genes, we found that many of the terms are related to ‘chromatin’, ‘G protein-coupled receptor activities’ and ‘RNA regulation’ (**Fig 4C** and **S3 Table**). These results are consistent with findings of Fru^M^ and *fru P1* roles for adult-specific functions [30,32]. Altogether, this suggests that there are dynamic gene expression changes occurring in these neurons in both sexes, as well as sex-specific gene expression which may contribute to sex differences in neuronal physiology in adults to generate male and female reproductive behaviors.

While the goal of this study was to gain insight into *fru P1* regulation of genes, the results presented can also serve as a launching point to further understand the functional roles of chromatin modifications and SEs. For example, we find that SEs do not always predict gene expression, suggesting that in some cases the SEs may mark a gene as poised for expression. The SE activity that directs gene expression may depend on the presence of other activating modifications that are found in enhancer regions, such as H3K4me1 [38]. Others have reported similar observations where computationally predicted enhancers are not necessarily linked to gene expression activity and show a dependence on transcription factor binding [83,84]. Furthermore, the Bachtrog laboratory also found that chromatin state did not account for sex-biased gene expression in studies performed at the larval stage [85]. Given that Fru^M^ has been implicated in recruiting chromatin modifiers, further direct Fru^M^ occupancy studies at these stages may elucidate its role in regulating gene expression through histone modifications. The application of machine learning approaches may also reveal new insights about the role of chromatin modifications in directing gene expression. Additional studies using techniques such as targeting chromatin modifying enzymes to particular loci, using Cas9-based approaches, can address the functional roles of the modifications presented here [86,87]. Moreover, *Drosophila* reproductive behaviors are an excellent model for studies examining how chromatin modifications and gene expression underlie behavioral plasticity, using models like long-term memory of courtship rejection and female post-mating behaviors [9,88]. The tools and the courtship model described here are a premiere system to go from high resolution genome-wide chromatin modifications and gene expression studies, to understanding the functional impacts of these molecular phenotypes on neuroanatomy and ultimately behavior.

## Materials and methods

### Chromotag fly constructs and strain generation

The H2B open reading frame was PCR amplified using high-fidelity Taq. The NsiI and SalI restriction enzyme sites were added to the 5’ and 3’ end, respectively, during PCR amplification, using the following oligonucleotides: atgcatgatATGCCTCCGAAAACTAGTGGAAAGGCAGCC and gtcgacTTATTTAGAGCTGGTGTACTTGGTGACAGCCTTGG. The resulting PCR product was Topo cloned (Invitrogen) and sequenced verified to ensure no PCR mutations were introduced. The H2B open reading frame was subsequently cloned into the p-UAS-3XFLAG-BLRP-mcherry-3’UTR plasmid for expression in *Drosophila*, using the Gal4/UAS system (plasmids provided by Paul Talbert and Steve Henikoff). The UAS-3XFLAG-BLRP-mcherry-H2B-3’UTR cassette was then cloned into the p2-Casp-UAS-BirA plasmid that contains the *E*. *coli* BirA gene under UAS control, P element ends and the mini-*white* gene (provided by Paul Talbert and Steve Henikoff). This construct was injected into *Drosophila white* strains for P element transformation by Genetivision Inc.

### *Drosophila* strains used in genomic assays

Flies with Chromatag expressed in *fru P1* neurons or broadly expressed with a pan-neuronal *elav* driver were the respective genotypes: *w;P[w*^*+mC*^, *UAS-Gal4]/P[UAS-Chromatag]*; *fru P1-Gal4/+* or *w;P[w*^*+mC*^, *UAS-Gal4]/P[UAS-Chromatag]*; *elav-Gal4/+*. Flies expressing the GFP-tagged variant of RpL10A in *fru P1* neurons were the genotype: *w/(w or* Y); *P[w*^*+mC*^, *UAS-Gal4]/P[w*^*+mC*^, *UAS-GFP*::*RpL10A]*; *fru P1-Gal4/+ [41].* The *elav-Gal4* strain was obtained from the Bloomington stock center. Flies were reared on standard cornmeal food at 25°C on a 12-h light/12-h dark cycle. List of fly stocks used in this study are reported in **S12 Table**.

### *Drosophila* tissue collection

For cell-type-specific ChIP-seq and TRAP-seq, approximately 1,000 sex-sorted flies were used. The time points were 48hr after pupal formation (APF, +/- 1hr), 16-24hrs post-eclosion, or 10–12 days post-eclosion. All flies were frozen at 1–2 hours after incubator lights turn on. All frozen animals were stored at -80°C until they were processed for downstream experiments. For the 48hr APF time point, flies were collected and sex sorted at the white prepupal stage, aged for two-days and snap frozen. For the 1-day time point, 0-8hrs post-eclosion flies were collected, aged to 16-24hrs for CO_2_ recovery and snap frozen. For the 10–12 day time point, bottles were cleared and adults were collected within two-days and sex sorted, transferred once to new vials while aging without additional CO_2_ anesthesia, and snap frozen.

### Translating ribosome affinity purification

TRAP mRNA purification was performed as previously described, with the following modifications [42]. At the last step, prior to library preparation the 40μl of lysate supernatant (input, total mRNA) and TRAP (IP) fractions were resuspended with RA1 lysis buffer to a total volume of 200μl for one additional column-based purification (Machery-Nagel Nucleospin RNA clean-up, product 7404948), using DNAse.

### Sequential ChIP

Four independent biological replicates were processed for each genotype and time point. Each biological replicate was divided into multiple samples, such that each replicate generated one input sample and samples to perform all histone modification ChIP analyses. Adult animals and pupae were snap frozen in liquid nitrogen and stored at -80°C (see above). The adult heads were separated from their bodies by vortexing frozen animals. The heads were sifted through liquid nitrogen-cooled mesh sieves #25 and #40 (with heads retained in the #40 sieve). The sequential ChIP methodology presented here is based on a previously published ChIP protocol [91]. Frozen adult heads or frozen whole body pupae (~0.3g) were dounce homogenized in 5ml of Buffer A1 (60mM KCL, 15mM NaCl, 4mM MgCl_2_, 15mM HEPES (pH 7.6), 0.5% TritonX-100, 0.5mM DTT, 10mM sodium butyrate, and Roche cOmplete Protease Inhibitor cocktail EDTA-free, product 5056489001), with 1% methanol-free formaldehyde (Pierce, 20% solution product 28906) for crosslinking at room temperature for 10 min. The crosslinking reaction was quenched using 540μl 2.5M Glycine and incubated for 5 minutes at room temperature.

Cross-linked homogenates were centrifuged at 4000g for 5 minutes at 4°C and the nuclear pellets were resuspended in Buffer A1. The nuclear pellets were washed 3 more times by centrifugation at 4000g for 5 minutes at 4°C with resuspension in 3ml of buffer A1. The nuclear pellets were washed an additional time with 1x Lysis Buffer without SDS (140mM NaCl, 15mM HEPES (pH 7.6), 1mM EDTA (pH 8.0), 0.5mM EGTA, 1% TritonX-100, 0.5mM DTT, 0.1% sodium deoxycholate, 10mM sodium butyrate, and Roche cOmplete Protease Inhibitor Cocktail EDTA-free) to remove all traces of Buffer A1 before the next lysis step. After one additional spin at 4000g for 5 minutes at 4°C, the washed nuclear pellet was resuspended in Lysis Buffer containing 1% SDS and 0.5% N-Laurosylsarcosine and incubated at 4°C, rotating for 1 hour. Nuclear lysates were quickly spun down for 10 seconds at 2000g and supernatant transferred to sonication tubes (Covaris; product 520130) for shearing. Chromatin was sheared for 25 minutes using a Covaris E220 (duty cycle: 5.0, peak incident power: 140, cycles per burst: 200). The sheared chromatin was transferred to microcentrifuge tubes and rotated for 10 min. at 4°C. Next, the sheared chromatin was centrifuged at 18,000g for 5 minutes at 16°C. The supernatant was transferred to a new microcentrifuge tube and the centrifugation step was repeated twice. The final supernatant was split into two 0.5ml aliquots (material from ~0.15g frozen tissue) and brought to a 1.5ml volume with 1x Lysis Buffer without SDS. The sheared chromatin was then aliquoted into 250μl volumes and stored at -80°C. To perform the sequential ChIP, 750μl of 1x Lysis Buffer without SDS was added to the 250μl of the thawed sheared chromatin. The sheared chromatin was incubated with 50μl of magnetic protein A/G beads (Pierce, product 88803) and 5μg of each respective antibody at 4°C overnight. The magnetic protein A/G beads were then washed four times with 1ml of Lysis Buffer wash (140mM NaCl, 15mM HEPES (pH 7.6), 1mM EDTA (pH 8.0), 0.5mM EGTA, 1% TritonX-100, 0.05% SDS, 0.5mM DTT, 0.1% sodium deoxycholate, 10mM sodium butyrate, and Roche cOmplete Protease Inhibitor Cocktail EDTA-free) and twice with TE (10mM Tris-HCL (pH 8.0), 1mM EDTA) for 10 minutes each, rotating at 4°C. Beads were incubated in 100μl ChIP elution buffer (100mM NaHCO_3_, 80mM NaOH, 1% SDS) at room temperature, rotating for 30–60 minutes to elute chromatin. The eluate was diluted 1:10 with Lysis Buffer without SDS and incubated on magnetic streptavidin beads (Invitrogen Dynabeads MyOne Streptavidin C1) for 4hrs-overnight rotating at 4°C. The beads were washed as above and chromatin was reverse crosslinked on beads in ChIP elution buffer (with 0.04mg/ml proteinase K, 0.01mg/ml RNaseA) at 65°C overnight. DNA was purified with a DNA purification and concentration kit (Machery-Nagel, product 740609).

### Illumina sequencing and library preparation

The isolated total RNA for TRAP libraries underwent poly(A)^+^ selection using the NEBNext Poly(A) mRNA isolation module (NEB, product E7490S). 48hr APF TRAP libraries were prepared with the NEBNext Ultra II Directional RNA Library Prep Kit for Illumina (NEB, product E7760S) and 10–12 day adult TRAP libraries were prepared with the NEBNext Ultra RNA library Prep Kit for Illumina (NEB, product E7530S). ChIP-seq libraries were prepared using the NEBNext ChIP-seq Library Prep Master Mix Set for Illumina (NEB, product E6240). Libraries were size selected using AMPure XP beads (Beckman) and clean up steps were performed using the Machery-Nagel PCR cleanup kit (Machery-Nagel, product 740609). For ChIP-seq and 48hr APF TRAP-seq, a final clean-up using AMpure XP beads was performed according to kit protocol to remove excess primers. Samples were pooled and sequenced on the Illumina HiSeq 2500 platform with 100-bp single end reads for 10–12 day adult TRAP libraries and 50-bp single end reads for ChIP-seq libraries. 48hr APF TRAP libraries were sequenced on the Illumina NovaSeq 6000 with 100-bp single end reads on an S1 flowcell.

### Sequencing read mapping

Read quality was assessed for each ChIP-seq and RNA-seq sequencing library with FastQC (v. 0.11.3). ChIP-seq reads were pre-processed using Trimmomatic’s ILLUMINACLIP (v. 0.33) option to remove Illumina adaptor sequences [92]. Pre-processed ChIP-seq reads were aligned to the *D*. *melanogaster* dm6 UCSC genome assembly [93], using Bowtie2 (v. 2.2.5), allowing up to 6 mismatches [94]. Only uniquely aligned reads were considered and individual replicate and pooled replicate (all replicates) BAM files were generated with SAMtools (v. 1.2) for downstream analyses [95]. Pairwise Spearman correlations between individual ChIP-seq replicates were performed in deepTools using the default “multiBamSummary” command and the “plotCorrelation” function using the “–CorMethod spearman” argument. Overall, we found high correlation between individual replicates retained in the analysis, with 97% of pairwise replicate comparisons having values >0.90 (**S1 Table**). There were 8 comparisons that had a lower correlation; however, no comparisons had a value lower than 0.80 (**S1 Table**). Based on early quality checks, we removed data generated from one of the four biological replicates for female *fru P1* 10–12 day conditions, resulting in n = 3 for all ChIP analyses for that genotype/time point. One statistical outlier for 1-day female *elav* H3K27ac was detected, resulting in an n = 3 for this condition (**S1 Table**). Sequencing coverage for pooled replicates for histone H3 and all histone modifications in this study were calculated using the pileup.sh function in BBMap on the uniquely mapped reads. RNA-seq reads were aligned using STAR (v. 2.7.2a) to the same genome assembly as previous [96].

### ChIP-seq peak calling and analysis

Regions of significant enrichment (peaks) for each histone modification (H3K27ac, H3K36me3, H3K4me3, H3K9me3, H3K27me3), at each time point (48hr APF, 1-day adult, 10–12 day adult), for each sex in *fru P1* or *elav* neurons, relative to sex and stage-specific input chromatin library controls for individual and the pooled replicates, were identified using the Model-based Analysis of ChIP-Seq, MACS2 (v.2.1.0) [55]. For each replicate, the peaks can be found in the GEO upload. For the pooled and consensus peaks see **S1–S10 Data**. We performed “narrow” peak calling for histone modifications with more compact enrichment patterns (H3K27ac and H3K4me3) and the “broad” peak option for modifications with diffuse enrichment (H3K36me3, H3K9me3, and H3K27me3) using the following: “callpeak” parameters:—gsize dm -p 1e-2—call-summits—fix-bimodal [97].

In an effort to resolve more peaks on the male X chromosome for all data sets we tried several modifications to our “callpeak” parameters by systematically adjusting—extsize,—bw, -q, and -p,—mfold, and–lambda. We also performed SICER peak calling, implemented through epic2, as an alternative peak calling approach. However, none of these changes to MACS2 or use of SICER achieved a sizeable increase in peaks called on the male X chromosome. Consensus peak sets among biological replicates were called using DiffBind requiring a peak to be present in 50% or more replicates [98]. MACS2 peak lists were annotated using the “annotatePeak” function in CHIPseeker, using default settings using the TxDb.Dmelanogaster.UCSC.dm6.ensGene database (v. 3.8) (peaks listed in **S1–S10 Data**) [99]. Annotations include a genomic feature assignment which indicates whether a peak is located in a TSS, exon, 5’ UTR, 3’ UTR, intronic, or intergenic region which was visualized for each experimental condition using the “plotAnnoBar function”.

### ChIP-seq correlation, visualization, and clustering analysis

Input normalized BigWig files for all histone modifications, replicates, and conditions were obtained using deepTools “bamCompare” function to normalize to input ChIP-seq read counts across the entire genome, over default 50bp bins with a log2 ratio summary of signal for each bin reported [52]. Using these normalized BigWig files, each experimental condition was compared using the “multiBigWigSummary” module to score ChIP-seq signal over 10kb windows (bins) across the genome and compressed into a matrix. These matrices were utilized in the “plotCorrelation” module for correlation analysis (Spearman correlation) to calculate similarity across histone modifications for both sexes, time points, and neuron types in our study (**S3 Fig**). To visualize normalized ChIP-seq signal over specified genomic regions of +/- 3kb of the TSS and TES, we used deepTools “computeMatrix” function (—beforeRegionStartLength 3000 –regionbodylength 5000—afterRegionStartlength 3000) and the “plotHeatmap” function to perform hierarchical clustering (ward linkage) and heatmaps were generated. Visual inspection of multiple clustering outputs was performed to select the optimal number of clusters in each analysis.

### RNA-seq analysis

Exon-level count tables were generated in FeatureCounts, for all replicates, using uniquely mapped reads for male and female *fru P1* TRAP and total mRNA for both 48hr APF and 10–12 day adult data sets [100]. Pairwise Spearman correlations between replicates for each condition were calculated in Microsoft Excel using the “RANK.AVE” and “CORREL” functions on counts-per-million values (CPM). The replicates were highly correlated (see **S6 Table)**. Exons were only retained in the analysis if they met our filtering criteria: exons that had counts above 2 CPM in 60% of the replicates for each pair-wise analysis. The pairwise comparisons are: 1) male TRAP vs. male total mRNA; 2) female TRAP vs. female total mRNA; 3) male total mRNA vs. female total mRNA; and 4) male TRAP vs. female TRAP. Filtered read counts were TMM-normalized and differential expression testing was performed on the exon level using edgeR’s Fisher’s exact testing for each pairwise comparison [101]. Significant differentially expressed exons were those with an FDR of <0.2 (Benjamini and Hochberg FDR-adjusted p value). All exons and associated FDR-adjusted p values are listed in **S6–S10 Tables**.

### Gene Ontology (GO) and pathway enrichment analyses

GO analysis and pathway enrichment analysis were conducted in R, using the clusterProfiler package, using a hypergeometric test for enrichment [102]. Additional GO analyses, protein domain, and pathway enrichment were carried out in Flymine [65].

### Immunohistochemistry and microscopy

Adult and 48hr APF brains were dissected in 1x Phosphate Buffered Saline (PBS; 140 mM NaCl, 10 mM phosphate buffer, and 3 mM KCl, pH 7.4) and fixed in 4% paraformaldehyde (Electron Microscopy Sciences, product 15713) in PBS at room temperature, for 20 minutes for adult brains, or 45 minutes for 48hr pupal brains and ventral nerve cords. Next, the brains were rinsed briefly 4 times with PBS and then washed three times with TNT (0.1M Tris-HCL, 0.3M NaCl, 0.5% Triton X-100), for 15 minutes for the first wash and 5 minutes for the two subsequent washes. The brains were blocked in Image-iT FX Signal Enhancer (Thermo Fisher) for 25 minutes and washed twice with TNT for 5 minutes each. Brains were incubated in TNT with primary antibody overnight at 4°C. After primary antibody was removed, the brains were washed six times with TNT for 5 minutes per wash. Next, the brains were incubated in TNT with secondary antibody for two hours at room temperature. The brains were then washed six times for 5 minutes in TNT, before being mounted in Vectashield mounting medium (Vector Laboratories, H-1000) on glass slides in Secureseal Image Spacers (Electron Microscopy Services) and covered with a no. 1.5 glass coverslip. Confocal microscopy was performed on a Zeiss LSM 700 system, with a Zeiss Plan-Apochromat 20x/0.8 M27 objective. A pinhole size of 1 Airy Unit (AU) was calculated in Zeiss Zen software (Black edition, 2012) as follows for each laser line: 488nm: 38μm; 555nm: 34μm; and 639nm: 39μm. Images were acquired at a resolution of 1024 × 1024 pixels using bidirectional scanning. The interval of each slice in the z-direction was set as 1.0 μm. Zeiss Zen software (Black edition, 2012) was used to make adjustments to laser power and detector gain to enhance the signal to noise ratio. Images were cropped using Adobe Photoshop CC 2018.

### Antibodies

The antibodies used for ChIP were: H3 (Abcam ab1791, lot no. GR177884-2), H3K27ac (Abcam ab4729, lot no. GR200563-1), H3K27me3 (Millipore 07–499, lot no. 2475696), H3K36me3 (Abcam ab9050, lot no. GR204353-1), H3K4me3 (Abcam ab8580, lot no. GR190202-1), and H3K9me3 (Abcam ab8898, lot no. GR186864-1), and were validated by the company for specificity. For the H3K4me3 antibody, abcam reports strong binding to H3K4me3 but some cross reactivity with H3K4me2 [103]. The following primary antibodies were used for immunofluorescence: mouse anti-Nc82 (1∶20; Developmental Studies Hybridoma Bank) and rat anti-Fru^M^ (1:200) [18]. The secondary antibodies used for immunofluorescence were Alexa Fluor goat anti-rat 488 (1:1000), goat anti-mouse 633 (1:500), rabbit anti-GFP 488 conjugate (1:600), and streptavidin 488 conjugate (1:4000) (Thermo Fisher).

### Behavior and viability assays

For courtship assays, all males were collected 0–6 hours post-eclosion and aged individually for 4–7 days at 25°C on a 12-h light/12-h dark cycle. The single male flies were either paired with a 4–7 day old *w; Canton S* (*CS*) virgin female (male-female) or male (male-male). Females were aged in groups of ~10 per vial. The assays were performed in a 10mm courtship chamber. Courtship activity was recorded for 10 min or until successful copulation at five-nine hours after fly incubator lights on, at 25°C (n = 12). Recordings were analyzed using Noldus software. Courtship Index (CI) and Wing Extension Index (WEI) were calculated by dividing the time the experimental male fly spent courting or unilaterally extending his wing toward the target female by the total observation time. JMP Pro 14 was used to conduct statistical analyses and statistical significance (p<0.05) was calculated using a nonparametric Kruskal-Wallis ANOVA test with Dunn’s post-hoc correction for courtship and wing extension index data. One-way ANOVA with Tukey post-hoc analysis was performed on copulation attempts data. The experimental male genotype is: *w; P[w*^*+mC*^, *UAS-Gal4]/P[UAS-Chromotag]*; *fru P1-Gal4/+*. The single transgene controls are: *w; P[w*^*+mC*^, *UAS-Gal4]/+*; *fru P1-Gal4/+*, and *w; P[UAS-Chromotag]*.

For locomotor activity, male and female flies of genotypes: *CS*, *w; P[w*^*+mC*^, *UAS-Gal4]/+*; *fru P1-Gal4/+*, and *w; P[UAS-Chromotag]*, and *w; P[w*^*+mC*^, *UAS-Gal4]/P[UAS-Chromotag]*; *fru P1-Gal4/+* were collected 0–6 hours post-eclosion, briefly anesthetized with CO_2_, loaded into glass tubes containing standard laboratory food, and tubes were mounted in *Drosophila* Activity Monitor (DAM) boards (Trikinetics, Waltham, MA). Data was collected in 5 minute bins and experiments were carried out at 25°C with a 12-h light/12-h dark cycle for 8 days. The first 24hrs of data was removed from the analysis, to recover CO_2_ and acclimate to the DAM system. Data analysis was performed with ShinyR-DAM [104] and R script analysis in R studio.

To determine if flies that express Chromatag ubiquitously are viable, we used the ubiquitous *daughterless-Gal4* (*da-Gal4*) driver to drive expression of *UAS-Chromatag*. Four independent crosses were set-up between w*; P[w^*+mW*.*hs*^ *= GAL4-da*.*G32]UH1*, *Sb*^*1*^*/TM6B*, *Tb*^*1*^ and *UAS-Chromatag*. The females laid eggs for five days. All progeny from each cross were counted and scored (**S2A Fig)**.

### Identification of enhancers and super-enhancers

Super-enhancer (SE) analyses were conducted using the ROSE algorithm: (https://bitbucket.org/young_computation/rose) [73,74]. Briefly, MACS2 H3K27ac peaks were stitched together, if within 12.5 kb of one another, for pooled replicates for each experimental condition. Regions +/- 2500bp to the TSS were excluded to remove promotor bias. The ROSE algorithm then determined the H3K27ac intensity inflexion point to determine typical enhancers versus super-enhancers (for all enhancers see **S11 Table**), as compared to input ChIP signal [73,74]. Genomic regions containing enhancers or super-enhancers were annotated using the default “annotatePeak” function in CHIPseeker [99]. H3K27ac SE-containing genes were compared to *fru P1* TRAP-enriched genes (defined above) and detected genes. A gene is considered detected if an exon passed our initial filter for the TRAP data sets but did not show significantly higher expression than total mRNA (**S6 and S7 Tables**). These criteria were also used for *elav* TRAP, but this analysis was conducted on the gene level instead of the exon level [41].

### Gene overlap and enrichment analysis

Venn diagrams to visualize overlapping genes were generated using online tools at http://bioinformatics.psb.ugent.be/webtools/Venn/ and size proportional Venn diagrams were made using BioVenn [105]. UpSet plots were generated using the UpSetR package [71]. Enrichment between gene lists was analyzed using the GeneOverlap R Package, which performs Fisher’s exact tests to determine whether enrichment is significant given genome size and reports a Jaccard index overlap and odds ratio [66]. All contingency tables reported in **S5 Table.**

## Supporting information

S1 Fig Experimental overview and visualization of *UAS-Chromatag* expression in *fru P1* neurons across time points. (**A**) Sequential ChIP-seq was performed in *fru P1* neurons from both sexes, at three time points: 48 hours after pupal formation (APF), 1-day adults (heads), and 10–12 day adults (heads) as well as in *elav-Gal4* expressing neurons in 1-day adult heads of both sexes. (**B-G**”) Anterior facing confocal maximum projections show *UAS*-*Chromatag* with no Gal4 driver in brains and ventral facing projections of ventral nerve cords (VNC) in females and males. (**B-B”**) 48hr APF female brains and (**C-C”**) VNCs, (**D-D”**) 10–12 day adult female brains, (**E-E”**) 48hr APF male brains and (**F-F”**) VNCs, and (**G-G”**) 10–12 day adult males brains. (**H-Q”**) Confocal maximum projections show *fru P1-Gal4* driven expression of *Chromatag*, which results in production of a tagged H2B variant that can be biotinylated. The H2B variant is produced and detected at all time points and in both sexes, used in this study (also see **Fig 1**). (**H-N”**) Biotinylated H2B is detected by fluor-conjugated streptavidin (cyan) in (**H-H”**) 48hr APF female brains and (**I-I”**) ventral nerve cords (VNC)s, (**J-J”**) 1-day adult female brains, (**K-K”**) 10–12 day adult female brains, (**L-L”**) 48hr APF male brains and (**M-M”**) VNCs, and (**N-N”**) 1-day adult males brains. (**O-Q”**) Fru^M^ is detected by immunostaining (green), using an anti-Fru^M^ antibody and mCherry tagged-H2B is visualized by genetically encoded mCherry in male (**O-O”**) 48hr APF brains and (**P-P”**) VNCs, and (**Q-Q”**) 1-day adult brains. Labels on 48hr APF brain images correspond to VNC images to right. Three-dimensional confocal maximum projections from successive confocal slices are presented with the most anterior slice positioned first. Brain and VNC anatomical axes are presented on lower left of first brain and VNC image panels: D, *dorsal*; M, *medial*; A, *anterior*; P, *posterior*; V, *ventral*. Further information on brain and VNC position and structure has been previously described [90,106]. Scale bars = 50 μm.
(PDF)

S2 Fig Locomotor activity and courtship behavior of males expressing *Chromatag* in *fru P1* neurons. (**A**) Numbers of viable progeny from each sex where *UAS-Chromatag* was crossed to *da-Gal4*, for four independent crosses. Cross number and progeny genotype are indicated along the bottom of graph. **(B)** Locomotor activity of males and females (n = 20–32 individuals per genotype). The average counts of beam crossing per individual, per day, is shown in boxplots, where upper and lower hinges correspond to the 25th and 75th percentiles (data collected using Drosophila Activity Monitors; Trikinetics). Activity data was analyzed by one-way ANOVA. Tukey HSD post-hoc tests were performed for each pairwise comparisons and significant differences are shown. **(C-I)** Male courtship toward a female (**C-F**), or male (**G-I**). The behavioral indices are the total time the male spends performing the courtship behavior/total video time, or the time until copulation occurs. (**C**) Male courtship index. (**E**) Wing extension index. (**E**) The number of copulation attempts toward the target female. (**F**) Percentage of males that successfully copulate with target females. (**G-I**) Male courtship toward *white Canton S* target male. (**G**) Male courtship index. (**H**) Wing extension index. (**I**) The number of copulation attempts toward the target male. (**C-I**) Horizontal bars show the mean (n = 12 males). Statistics performed for all pair-wise comparisons. Kruskal-Wallis ANOVA test with Dunn’s post-hoc correction used in tests with courtship and wing extension index data. One-way ANOVA with Tukey post-hoc analysis performed on copulation attempts data. Significance levels are indicated: p<0.05 *, p<0.01 **, p<0.001 ***, p<0.0001 ****.
(PDF)

S3 Fig Spearman correlation of Chromatag-ChIP data sets. (**A**) The positional correlation of genome-wide ChIP-seq signals from all the data sets, comparing 10kb bins, using unsupervised hierarchical clustering is shown. The level of positive or negative correlation is indicated by color (see scale bar). The dendrogram on the left indicates which samples read positions are most similar to each other. Additional color coding by groups with positive correlation is presented. For each chromatin data set, the sex, H3 modification, time point and neuronal cell type is indicated. This analysis was performed using the pooled replicates for each data set (n = 3–4). Abbreviations for sex, stage, and H3 modifications are: Male (M), Female (F), 48hr APF (48P), 1-day adult (1A),10–12 day adult (10A), H3K27ac (27ac), H3K27me3 (27me3), H3K36me3 (36me3), H3K4me3 (4me3), and H3K9me3 (9me3).
(PDF)

S4 Fig Genomic feature distribution of MACS2 consensus peaks. **(A-J)** Genomic feature distributions of MACS2 consensus peaks, those occurring in two or more biological replicates, for activating (H3K27ac, H3K36me3, H3K4me3; purple) and repressive (H3K27me3 and H3K9me3; black) H3 chromatin modifications. The histone modification is indicated on the left. Each panel includes data for *fru P1* (black) and *elav* neurons (gray) in males (left) and females (right). The X-axis shows the percent of each genomic feature defined in the legend (bottom). All MACS2 peaks in this analysis are listed in **S9 and S10 Data**. Abbreviations: 5’ untranslated region (5’ UTR) and 3’ untranslated region (3’ UTR).
(PDF)

S5 Fig Overlap of genes containing MACS2 peaks and genes with bivalent promoters across time points in *fru P1* neurons. **(A-J)** Venn diagrams comparing genes that have at least one MACS2 peak for each chromatin modification, across time points and within sex. For each Venn diagram category, the number of genes and the proportion of the total genes (in parentheses) in each panel is shown. The histone modification is indicated on the left (activating in purple, repressive in black). The legend for each time point is on the top left and male and female data sets are indicated at the top. All MACS2 peaks were called on pooled biological replicates (n = 3–4) and identified as enriched relative to matched input controls. (**K**) Venn diagrams for genes with bivalent promoter regions (containing both H3K4me3 and H3K27me3) at each time point in *fru P1* neurons in males (left) and females (right). MACS2 peaks and genes from Venn diagram analyses are listed in **S1–S3 Data, S5–S7 Data** and **S2 Table**.
(PDF)

S6 Fig GO for genes with modifications that persist across all three time points in *fru P1* neurons. (**A-B**) Gene Ontology (GO) enrichment analysis for genes with modifications that persist across all three time points in *fru P1* neurons in (**A**) males and (**B**) females (centers of Venn diagrams in **S5 Fig**). The GO categories are molecular function, biological process, and cellular component. The GO terms shown in the plots are the top ten most significantly enriched terms for each list (non-redundant shown; Benjamini-Hochberg, p<0.05). The size of each dot indicates the number of genes (count) and the color indicates the p value (p.adjust). The GO information is in **S3 Table** and gene lists are in **S2 Table**.
(PDF)

S7 Fig TRAP sequencing read coverage of *fruitless* in 48hr APF and 10–12 day data sets and analysis of sex-biased TRAP genes. **(A)** Average read coverage in 48hr APF TRAP and (**B**) 10–12 day adult TRAP for males (blue) and females (red) across the *fruitless* locus. The 5’ end of *fruitless* is located on the right and transcription runs in the direction of the arrows on the gene structure (right to left). **(C)** Sex-biased TRAP genes were identified by comparing expression between the male and female TRAP samples. For a gene to be considered sex-biased in the TRAP comparison, at least one exon needed to be more highly expressed in the TRAP sample of one sex (FDR<0.2). Comparison of male-biased TRAP genes (teal) and female-biased TRAP genes (purple) for all time points examined. For each Venn diagram category, the number of genes and the proportion of the total genes (in parentheses) in each panel is shown. (**D-E**) Venn diagrams comparing sex-biased TRAP genes across time points within each sex. (**F**) Upset intersection plot of sex-biased TRAP genes from 48hr APF (blue), 1-day adult (red) [42] and 10–12 day adult (green) time points [71]. n = 4–5 biological replicates per condition. (**G**) Gene Ontology (GO) enrichment analysis for sex-biased TRAP genes. The GO categories are molecular function, biological process, and cellular component. The GO terms shown in the plots are the top ten most significantly enriched terms for each list (non-redundant shown; Benjamini-Hochberg, p<0.05). The size of each dot indicates the number of genes (count) and the color indicates the p value (p.adjust). The GO information is in **S3 Table** and gene lists are in **S9 and S10 Tables**.
(PDF)

S8 Fig Hierarchical clustering of histone modification distributions for elav TRAP-enriched genes. For detailed description of heatmaps see **Fig 3**. Heatmaps for *elav* chromatin data from 1-day adults based on *elav* TRAP data enrichments [41]. **(A-B)** Heatmap for genes that are TRAP-enriched in 1-day adult *elav* neurons. **(C-D)** Heatmap for genes enriched in total mRNA. For each cluster of genes, the average fold-enrichment (Avg FC) of gene expression, indicated on the left (TRAP-enrichment), was calculated. For each cluster of genes, the average expression is indicated on the right for the TRAP and total mRNA data. Average fold-enrichment in gene expression and average expression in fragments per kilobase per million (FPKM) was calculated per cluster. The average was from expression data for genes that were significantly enriched in *elav* neurons (TRAP) from both sexes, or whole head tissue (total mRNA), averaged over both sexes (FDR<0.2; [41]). Gene lists for each cluster are provided in **S4 Table**.
(PDF)

S9 Fig Hierarchical clustering of histone modification distributions for total mRNA-enriched genes. Hierarchal clustering reveals shared combinations of histone modifications for total mRNA-enriched genes relative to TRAP, generated using deepTools. For detailed description of heatmaps see **Fig 3**. Heatmaps for *fru P1* chromatin data from 48hr APF **(A-B)**, 1-day adults **(C-D)**, and 10–12 day adults **(E-F)**. Gene lists for each cluster are provided in **S4 Table**. For each cluster of genes, the average fold-enrichment (Avg FC) of gene expression is indicated on the left (total mRNA-enrichment), calculated at the exon level. For each cluster of genes, the average expression is indicated on the right for the TRAP and total mRNA data, calculated at the exon level. The 48hr APF and 10–12 day adult gene expression levels are provided as reads per million (RPM). The 1-day adult time point gene expression levels are provided as reads per kilobase per million (RPKM) [42]. For TRAP and total mRNA expression data see **S6–S8 Tables**.
(PDF)

S10 Fig Hierarchical clustering of histone modification distributions for TRAP-detected genes. Hierarchal clustering reveals shared combinations of histone modifications for genes detected in expression data sets, generated using deepTools. For detailed description of heatmaps see **Fig 3**. Heatmaps for *fru P1* chromatin data from 48hr APF **(A-B)**, 1-day adults **(C-D)**, and 10–12 day adults **(E-F)**. A gene was considered detected in our 48hr APF or 10–12 day adult TRAP libraries if one or more exons were detected based on our initial filtering criteria (see Materials and Methods). Genes detected in 1-day adult TRAP were those identified in [42]. Gene lists for each cluster are provided in **S4 Table**.
(PDF)

S11 Fig Super-enhancers identified based on H3K27ac peaks in *fru P1*- and *elav* neuron chromatin data sets for autosomes. **(A)** Venn diagrams showing the overlap between *fru P1* SE-containing genes identified on autosomes across time points for males (left) and females (right). **(B)** Percentages of SE-containing genes for each stage and neuron type (*fru P1* or *elav*) that are male-specific, female-specific or occur in both sexes.
(PDF)

S1 Table Replicate correlation, sequencing coverage and genes with bivalent promoters. Excel data table with 4 sheets containing the correlations of all ChIP-seq replicates, sequencing coverage, and lists of genes with bivalent promoters.
(XLSX)

S2 Table Genes represented in Venn diagrams showing overlap of genes containing MACS2 peaks across time points in *fru P1* and *elav* neurons. Excel data table with 21 sheets containing Venn diagram gene matrices for Figs 2 and S5.
(XLSX)

S3 Table All Gene Ontology (GO) results. Excel data table containing 44 sheets with all GO enrichment results.
(XLSX)

S4 Table All heatmap cluster gene matrices. Excel data table with 5 sheets containing gene matrices for all heatmaps in Figs 3, 5, S8, S9 and S10.
(XLSX)

S5 Table All gene list overlap testing. Excel data table with 7 sheets containing all overlap testing results from analyses performed using the GeneOverlap package in R.
(XLSX)

S6 Table Differentially expressed exons and genes between 48hr APF *fru P1* TRAP and total mRNA. Excel data table with 16 sheets containing the replicate correlations, and all EdgeR outputs for all exon level differential expression testing between sex-specific 48hr APF *fru P1* TRAP and total mRNA.
(XLSB)

S7 Table Differentially expressed exons and genes between 10–12 day adult *fru P1* TRAP and total mRNA. Excel data table with 16 sheets containing the replicate correlations, and all EdgeR outputs for all exon level differential expression testing between sex-specific 10–12 day adult *fru P1* TRAP and total mRNA.
(XLSB)

S8 Table Differentially expressed exons genes with higher expression in total mRNA. Excel data table with 8 sheets containing all EdgeR outputs for all exon level differential expression testing between sex-specific 48hr APF and 10–12 day adult ***fru P1*** TRAP and total mRNA.
(XLSX)

S9 Table Differentially expressed exons and genes between 48hr APF male and female *fru P1* TRAP and total mRNA. Excel data table with 14 sheets containing all EdgeR outputs for all exon level differential expression testing between 48hr APF male and female *fru P1* TRAP and total mRNA.
(XLSB)

S10 Table Differentially expressed exons and genes between 10–12 day adult male and female *fru P1* TRAP and total mRNA. Excel data table with 14 sheets containing all EdgeR outputs for all exon level differential expression testing between 10–12 day adult male and female *fru P1* TRAP and total mRNA.
(XLSX)

S11 Table Enhancers and super-enhancers with gene annotations and expression information. Excel data table with 21 sheets containing all enhancer and super enhancer data.
(XLSX)

S12 Table Complete genotypes of *Drosophila* strains used in this study. Excel data table of all *Drosophila* strains used in this study.
(XLSX)

S1 Data 48hr APF male MACS2 peak data for all modifications in *fru P1* neurons. Annotated MACS2 peaks for all pooled biological replicates for all modifications in *fru P1* neurons in males at 48hr APF.
(XLSB)

S2 Data 1-day adult male MACS2 peak data for all modifications in *fru P1* neurons. Annotated MACS2 peaks for all pooled biological replicates for all modifications in *fru P1* neurons in males in 1-day adults.
(XLSB)

S3 Data 10–12 day adult male MACS2 peak data for all modifications in *fru P1* neurons. Annotated MACS2 peaks for all pooled biological replicates for all modifications in *fru P1* neurons in males in 10–12 day adults.
(XLSB)

S4 Data 1-day adult male MACS2 peak data for all modifications in *elav* neurons. Annotated MACS2 peaks for all pooled biological replicates for all modifications in *elav* neurons in males in 1-day adults.
(XLSB)

S5 Data 48hr APF female MACS2 peak data for all modifications in *fru P1* neurons. Annotated MACS2 peaks for all pooled biological replicates for all modifications in *fru P1* neurons in females at 48hr APF.
(XLSB)

S6 Data 1-day adult female MACS2 peak data for all modifications in *fru P1* neurons. Annotated MACS2 peaks for all pooled biological replicates for all modifications in *fru P1* neurons in females in 1-day adults.
(XLSB)

S7 Data 10–12 day adult female MACS2 peak data for all modifications in *fru P1* neurons. Annotated MACS2 peaks for all pooled biological replicates for all modifications in *fru P1* neurons in females in 10–12 day adults.
(XLSB)

S8 Data 1-day adult female MACS2 peak data for all modifications in *elav* neurons. Annotated MACS2 peaks for all pooled biological replicates for all modifications in *elav* neurons in females in 1-day adults.
(XLSB)

S9 Data Annotated consensus MACS2 peaks for all modifications in male *fru P1* neurons. Annotated consensus peaks obtained from Diffbind across biological replicates for all modifications in male *fru P1* neurons.
(XLSB)

S10 Data Annotated consensus MACS2 peaks for all modifications in female *fru P1* neurons. Annotated consensus peaks obtained from Diffbind across biological replicates for all modifications in female *fru P1* neurons.
(XLSB)
