# Supplementary material for: Analysis of cell-type-specific chromatin modifications and gene expression in Drosophila neurons that direct reproductive behavior
Source: PLoS Genet. 2021 Apr 26;17(4):e1009240. doi: 10.1371/journal.pgen.1009240 (PMC8102012; doi:10.1371/journal.pgen.1009240)
Supplement: S1 Fig — (A) Sequential ChIP-seq was performed in fru P1 neurons from both sexes, at three time points: 48 hours after pupal formation (APF), 1-day adults (heads), and 10–12 day adults (heads) as well as in elav-Gal4 expressing neurons in 1-day adult heads of both sexes. (B-G”) Anterior facing confocal maximum projections show UAS-Chromatag with no Gal4 driver in brains and ventral facing projections of ventral nerve cords (VNC) in females and males. (B-B”) 48hr APF female brains and (C-C”) VNCs, (D-D”) 10–12 day adult female brains, (E-E”) 48hr APF male brains and (F-F”) VNCs, and (G-G”) 10–12 day adult males brains. (H-Q”) Confocal maximum projections show fru P1-Gal4 driven expression of Chromatag, which results in production of a tagged H2B variant that can be biotinylated. The H2B variant is produced and detected at all time points and in both sexes, used in this study (also see Fig 1). (H-N”) Biotinylated H2B is detected by fluor-conjugated streptavidin (cyan) in (H-H”) 48hr APF female brains and (I-I”) ventral nerve cords (VNC)s, (J-J”) 1-day adult female brains, (K-K”) 10–12 day adult female brains, (L-L”) 48hr APF male brains and (M-M”) VNCs, and (N-N”) 1-day adult males brains. (O-Q”) FruM is detected by immunostaining (green), using an anti-FruM antibody and mCherry tagged-H2B is visualized by genetically encoded mCherry in male (O-O”) 48hr APF brains and (P-P”) VNCs, and (Q-Q”) 1-day adult brains. Labels on 48hr APF brain images correspond to VNC images to right. Three-dimensional confocal maximum projections from successive confocal slices are presented with the most anterior slice positioned first. Brain and VNC anatomical axes are presented on lower left of first brain and VNC image panels: D, dorsal; M, medial; A, anterior; P, posterior; V, ventral. Further information on brain and VNC position and structure has been previously described [90,106]. Scale bars = 50 μm. (PDF) [file pgen.1009240.s001.pdf]

A

Neuron Type	Time-points profiled
fru P1	48hr APF, 1-day adult, 10-12 day adult
elav	1-day adult

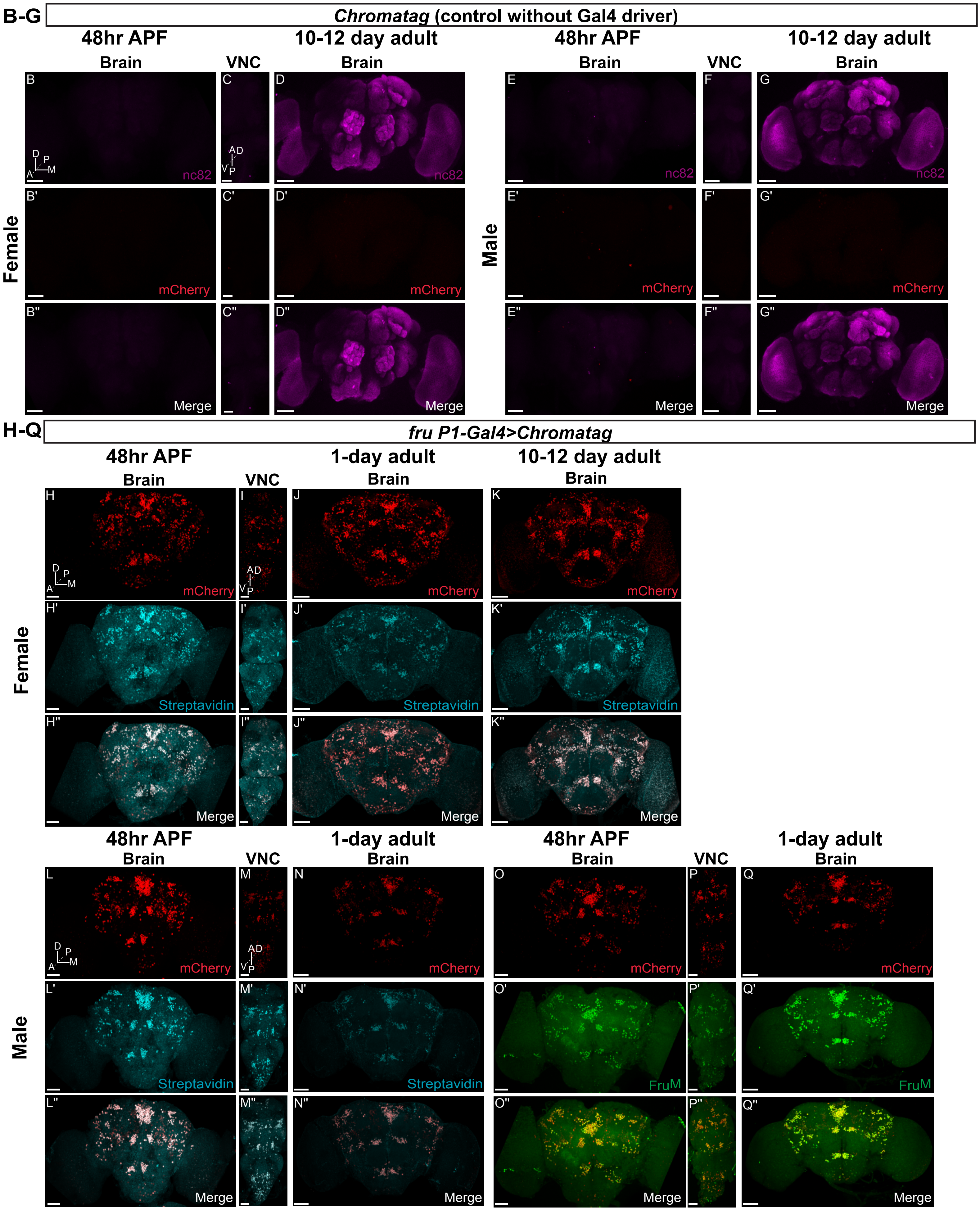

S1 Fig. Experimental overview and visualization of *UAS-Chromatag* expression in *fru P1* neurons across time points. (**A**) Sequential ChIP-seq was performed in *fru P1* neurons from both sexes, at three time points: 48 hours after pupal formation (APF), 1-day adults (heads), and 10-12 day adults (heads) as well as in *elav-Gal4* expressing neurons in 1-day adult heads of both sexes. (**B-G**") Anterior facing confocal maximum projections show *UAS-Chromatag* with no Gal4 driver in brains and ventral facing projections of ventral nerve cords (VNC) in females and males. (**B-B"**) 48hr APF female brains and (**C-C"**) VNCs, (**D-D"**) 10-12 day adult female brains, (**E-E"**) 48hr APF male brains and (**F-F"**) VNCs, and (**G-G"**) 10-12 day adult males brains. (**H-Q"**) Confocal maximum projections show *fru P1-Gal4* driven expression of *Chromatag*, which results in production of a tagged H2B variant that can be biotinylated. The H2B variant is produced and detected at all time points and in both sexes, used in this study (also see **Fig 1**). (**H-N"**) Biotinylated H2B is detected by fluor-conjugated streptavidin (cyan) in (**H-H"**) 48hr APF female brains and (**I-I"**) ventral nerve cords (VNC)s, (**J-J"**) 1-day adult female brains, (**K-K"**) 10-12 day adult female brains, (**L-L"**) 48hr APF male brains and (**M-M"**) VNCs, and (**N-N"**) 1-day adult males brains. (**O-Q"**) Fru^M is detected by immunostaining (green), using an anti-Fru^M antibody and mCherry tagged-H2B is visualized by genetically encoded mCherry in male (**O-O"**) 48hr APF brains and (**P-P"**) VNCs, and (**Q-Q"**) 1-day adult brains. Labels on 48hr APF brain images correspond to VNC images to right. Three-dimensional confocal maximum projections from successive confocal slices are presented with the most anterior slice positioned first. Brain and VNC anatomical axes are presented on lower left of first brain and VNC image panels: D, *dorsal*; M, *medial*; A, *anterior*; P, *posterior*; V, *ventral*. Further information on brain and VNC position and structure has been previously described [90,106]. Scale bars = 50 μm.
