# Supplementary material for: Analysis of cell-type-specific chromatin modifications and gene expression in Drosophila neurons that direct reproductive behavior
Source: PLoS Genet. 2021 Apr 26;17(4):e1009240. doi: 10.1371/journal.pgen.1009240 (PMC8102012; doi:10.1371/journal.pgen.1009240)
Supplement: S2 Fig — (A) Numbers of viable progeny from each sex where UAS-Chromatag was crossed to da-Gal4, for four independent crosses. Cross number and progeny genotype are indicated along the bottom of graph. (B) Locomotor activity of males and females (n = 20–32 individuals per genotype). The average counts of beam crossing per individual, per day, is shown in boxplots, where upper and lower hinges correspond to the 25th and 75th percentiles (data collected using Drosophila Activity Monitors; Trikinetics). Activity data was analyzed by one-way ANOVA. Tukey HSD post-hoc tests were performed for each pairwise comparisons and significant differences are shown. (C-I) Male courtship toward a female (C-F), or male (G-I). The behavioral indices are the total time the male spends performing the courtship behavior/total video time, or the time until copulation occurs. (C) Male courtship index. (E) Wing extension index. (E) The number of copulation attempts toward the target female. (F) Percentage of males that successfully copulate with target females. (G-I) Male courtship toward white Canton S target male. (G) Male courtship index. (H) Wing extension index. (I) The number of copulation attempts toward the target male. (C-I) Horizontal bars show the mean (n = 12 males). Statistics performed for all pair-wise comparisons. Kruskal-Wallis ANOVA test with Dunn’s post-hoc correction used in tests with courtship and wing extension index data. One-way ANOVA with Tukey post-hoc analysis performed on copulation attempts data. Significance levels are indicated: p<0.05 *, p<0.01 **, p<0.001 ***, p<0.0001 ****. (PDF) [file pgen.1009240.s002.pdf]

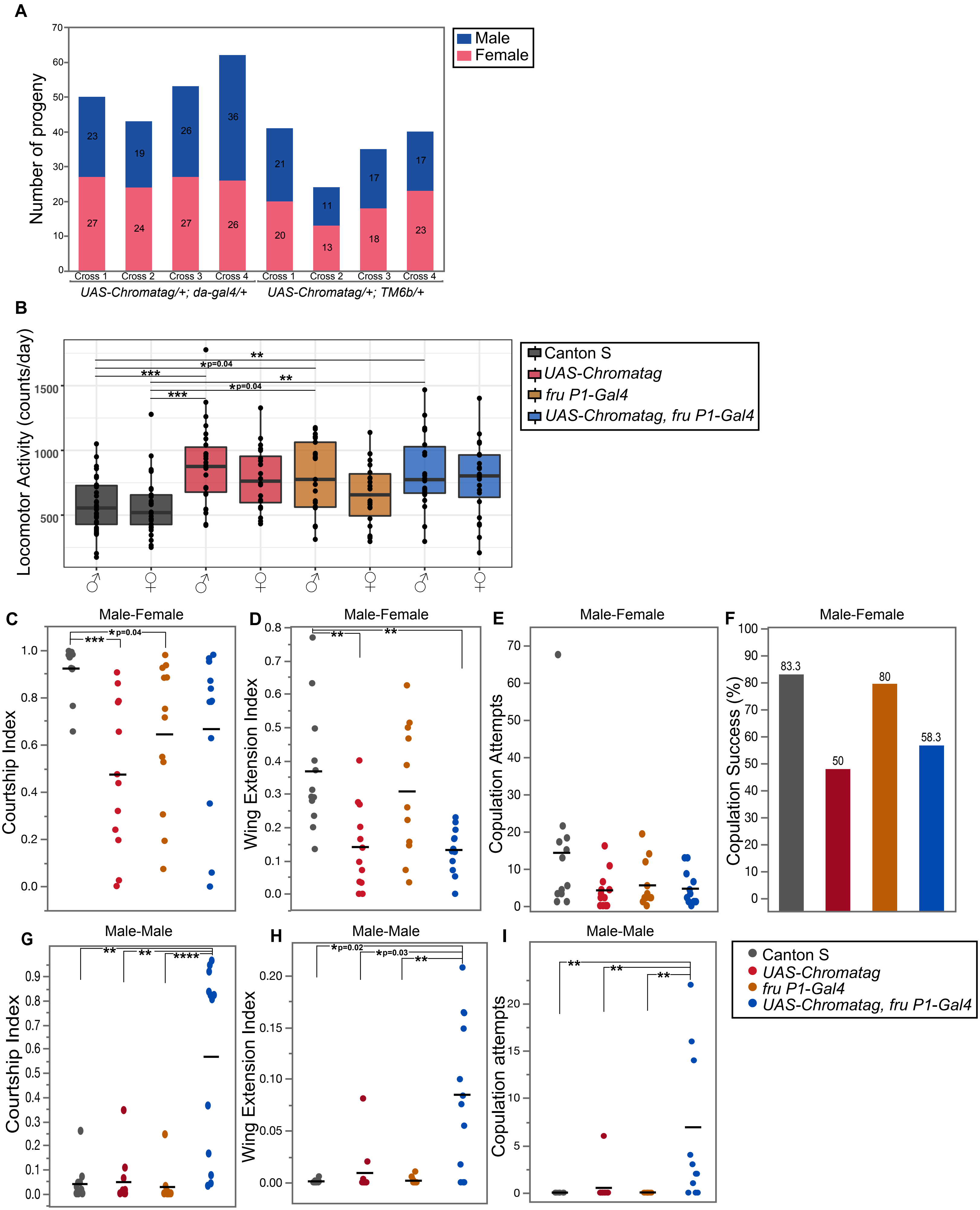

A
Number of progeny
Male
Female
Cross 1
Cross 2
Cross 3
Cross 4
UAS-Chromatag/+; da-gal4/+
UAS-Chromatag/+; TM6b/+
B
Locomotor Activity (counts/day)
Canton S
UAS-Chromatag
fru P1-Gal4
UAS-Chromatag, fru P1-Gal4
*p=0.04
C
Male-Female
Courtship Index
D
Male-Female
Wing Extension Index
E
Male-Female
Copulation Attempts
F
Male-Female
Copulation Success (%)
83.3
50
80
58.3
G
Male-Male
Courtship Index
H
Male-Male
Wing Extension Index
*p=0.02
*p=0.03
I
Male-Male
Copulation attempts
Canton S
UAS-Chromatag
fru P1-Gal4
UAS-Chromatag, fru P1-Gal4

S2 Fig. Locomotor activity and courtship behavior of males expressing *Chromatag* in *fru P1* neurons. (**A**) Numbers of viable progeny from each sex where *UAS-Chromatag* was crossed to *da-Gal4*, for four independent crosses. Cross number and progeny genotype are indicated along the bottom of graph. **(B)** Locomotor activity of males and females (n=20-32 individuals per genotype). The average counts of beam crossing per individual, per day, is shown in boxplots, where upper and lower hinges correspond to the 25th and 75th percentiles (data collected using Drosophila Activity Monitors; Trikinetics). Activity data was analyzed by one-way ANOVA. Tukey HSD post-hoc tests were performed for each pairwise comparisons and significant differences are shown. **(C-I)** Male courtship toward a female (**C-F**), or male (**G-I**). The behavioral indices are the total time the male spends performing the courtship behavior/total video time, or the time until copulation occurs. (**C**) Male courtship index. (**E**) Wing extension index. (**E**) The number of copulation attempts toward the target female. (**F**) Percentage of males that successfully copulate with target females. (**G-I**) Male courtship toward *white Canton S* target male. (**G**) Male courtship index. (**H**) Wing extension index. (**I**) The number of copulation attempts toward the target male. (**C-I**) Horizontal bars show the mean (n=12 males). Statistics performed for all pair-wise comparisons. Kruskal-Wallis ANOVA test with Dunn's post-hoc correction used in tests with courtship and wing extension index data. One-way ANOVA with Tukey post-hoc analysis performed on copulation attempts data. Significance levels are indicated: p<0.05 *, p<0.01 **, p<0.001 ***, p<0.0001 ****.
