# Supplementary material for: Analysis of cell-type-specific chromatin modifications and gene expression in Drosophila neurons that direct reproductive behavior
Source: PLoS Genet. 2021 Apr 26;17(4):e1009240. doi: 10.1371/journal.pgen.1009240 (PMC8102012; doi:10.1371/journal.pgen.1009240)
Supplement: S3 Fig — (A) The positional correlation of genome-wide ChIP-seq signals from all the data sets, comparing 10kb bins, using unsupervised hierarchical clustering is shown. The level of positive or negative correlation is indicated by color (see scale bar). The dendrogram on the left indicates which samples read positions are most similar to each other. Additional color coding by groups with positive correlation is presented. For each chromatin data set, the sex, H3 modification, time point and neuronal cell type is indicated. This analysis was performed using the pooled replicates for each data set (n = 3–4). Abbreviations for sex, stage, and H3 modifications are: Male (M), Female (F), 48hr APF (48P), 1-day adult (1A),10–12 day adult (10A), H3K27ac (27ac), H3K27me3 (27me3), H3K36me3 (36me3), H3K4me3 (4me3), and H3K9me3 (9me3). (PDF) [file pgen.1009240.s003.pdf]

A

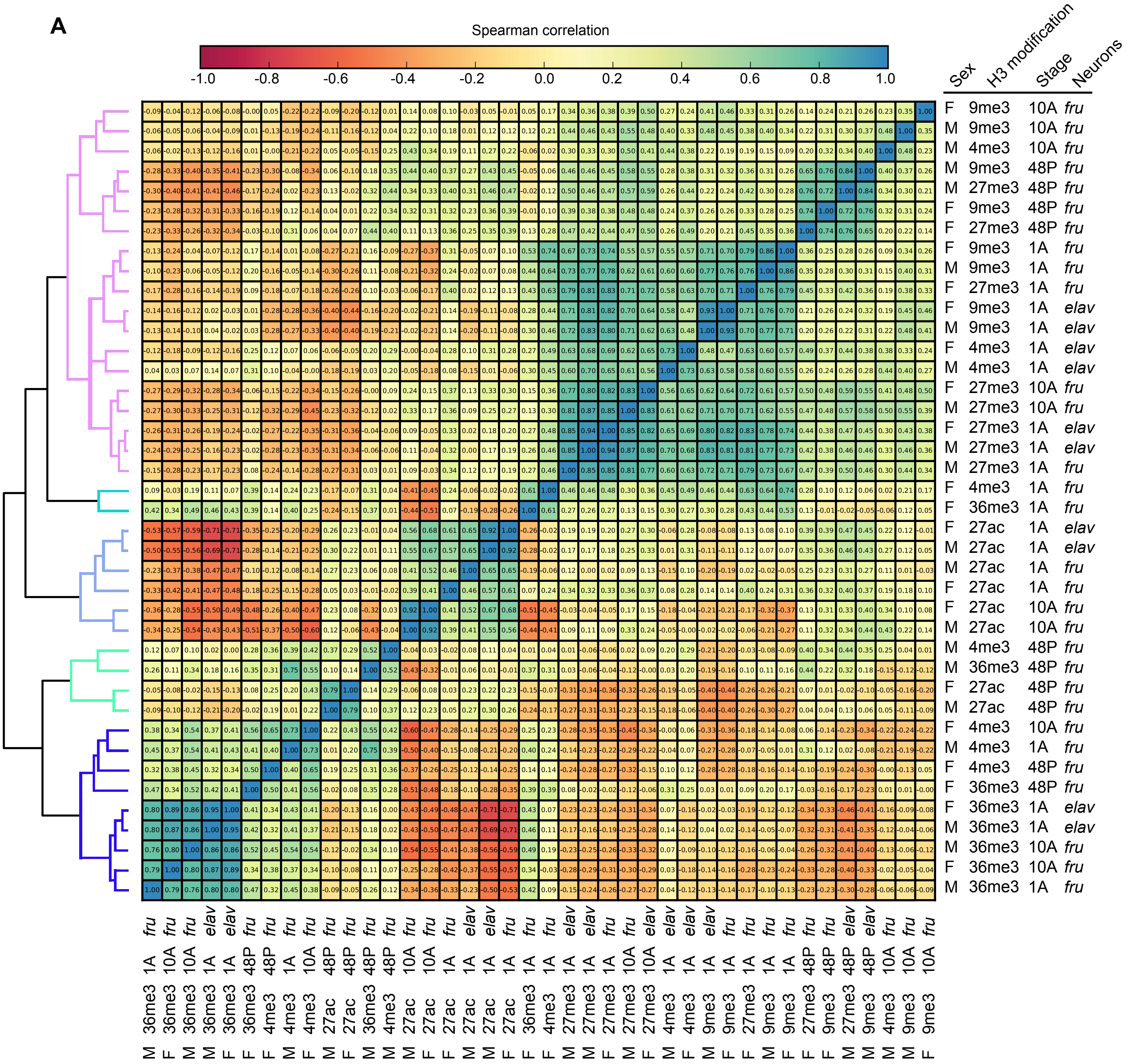
Spearman correlation
-1.0
-0.8
-0.6
-0.4
-0.2
0.0
0.2
0.4
0.6
0.8
1.0
Sex
H3 modification
Stage
Neurons
F 9me3 10A fru
M 9me3 10A fru
M 4me3 10A fru
M 9me3 48P fru
M 27me3 48P fru
F 9me3 48P fru
F 27me3 48P fru
F 9me3 1A fru
M 9me3 1A fru
F 27me3 1A fru
F 9me3 1A elav
M 9me3 1A elav
F 4me3 1A elav
M 4me3 1A elav
F 27me3 10A fru
M 27me3 10A fru
F 27me3 1A elav
M 27me3 1A elav
M 27me3 1A fru
F 4me3 1A fru
F 36me3 1A fru
F 27ac 1A elav
M 27ac 1A elav
M 27ac 1A fru
F 27ac 1A fru
F 27ac 10A fru
M 27ac 10A fru
M 4me3 48P fru
M 36me3 48P fru
F 27ac 48P fru
M 27ac 48P fru
F 4me3 10A fru
M 4me3 1A fru
F 4me3 48P fru
F 36me3 48P fru
F 36me3 1A elav
M 36me3 1A elav
M 36me3 10A fru
F 36me3 10A fru
M 36me3 1A fru

S3 Fig. Spearman correlation of Chromatag-ChIP data sets. (**A**) The positional correlation of genome-wide ChIP-seq signals from all the data sets, comparing 10kb bins, using unsupervised hierarchical clustering is shown. The level of positive or negative correlation is indicated by color (see scale bar). The dendrogram on the left indicates which samples read positions are most similar to each other. Additional color coding by groups with positive correlation is presented. For each chromatin data set, the sex, H3 modification, time point and neuronal cell type is indicated. This analysis was performed using the pooled replicates for each data set (n=3-4). Abbreviations for sex, stage, and H3 modifications are: Male (M), Female (F), 48hr APF (48P), 1-day adult (1A),10-12 day adult (10A), H3K27ac (27ac), H3K27me3 (27me3), H3K36me3 (36me3), H3K4me3 (4me3), and H3K9me3 (9me3).
