# Supplementary material for: Analysis of cell-type-specific chromatin modifications and gene expression in Drosophila neurons that direct reproductive behavior
Source: PLoS Genet. 2021 Apr 26;17(4):e1009240. doi: 10.1371/journal.pgen.1009240 (PMC8102012; doi:10.1371/journal.pgen.1009240)
Supplement: S4 Fig — (A-J) Genomic feature distributions of MACS2 consensus peaks, those occurring in two or more biological replicates, for activating (H3K27ac, H3K36me3, H3K4me3; purple) and repressive (H3K27me3 and H3K9me3; black) H3 chromatin modifications. The histone modification is indicated on the left. Each panel includes data for fru P1 (black) and elav neurons (gray) in males (left) and females (right). The X-axis shows the percent of each genomic feature defined in the legend (bottom). All MACS2 peaks in this analysis are listed in S9 and S10 Data. Abbreviations: 5’ untranslated region (5’ UTR) and 3’ untranslated region (3’ UTR). (PDF) [file pgen.1009240.s004.pdf]

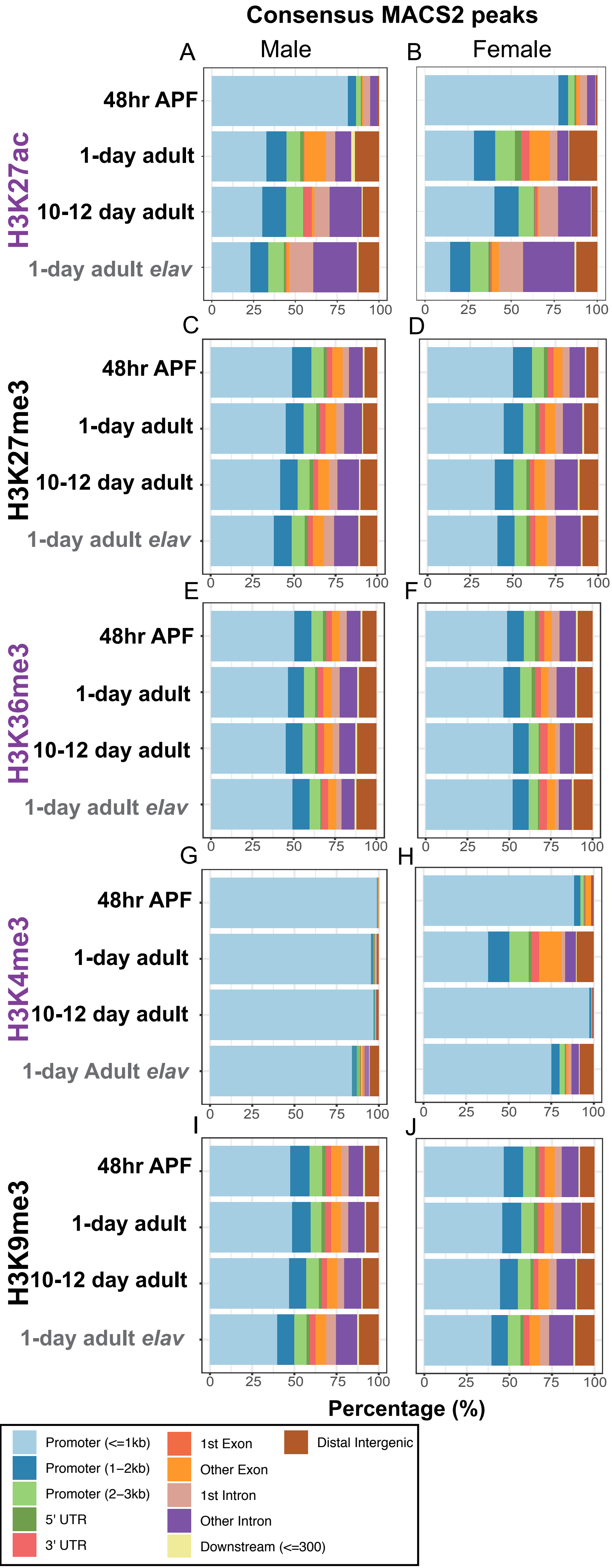

Consensus MACS2 peaks
A
Male
B
Female
H3K27ac
H3K27me3
H3K36me3
H3K4me3
H3K9me3
C
D
E
F
G
H
I
J
48hr APF
1-day adult
10-12 day adult
1-day adult *elav*
1-day Adult *elav*
0
25
50
75
100
Percentage (%)
Promoter (<=1kb)
Promoter (1–2kb)
Promoter (2–3kb)
5' UTR
3' UTR
1st Exon
Other Exon
1st Intron
Other Intron
Downstream (<=300)
Distal Intergenic

S4 Fig. Genomic feature distribution of MACS2 consensus peaks. **(A-J)** Genomic feature distributions of MACS2 consensus peaks, those occurring in two or more biological replicates, for activating (H3K27ac, H3K36me3, H3K4me3; purple) and repressive (H3K27me3 and H3K9me3; black) H3 chromatin modifications. The histone modification is indicated on the left. Each panel includes data for *fru P1* (black) and *elav* neurons (gray) in males (left) and females (right). The X-axis shows the percent of each genomic feature defined in the legend (bottom). All MACS2 peaks in this analysis are listed in **S9-S10 Data**. Abbreviations: 5' untranslated region (5' UTR) and 3' untranslated region (3' UTR).
