# Supplementary material for: Analysis of cell-type-specific chromatin modifications and gene expression in Drosophila neurons that direct reproductive behavior
Source: PLoS Genet. 2021 Apr 26;17(4):e1009240. doi: 10.1371/journal.pgen.1009240 (PMC8102012; doi:10.1371/journal.pgen.1009240)
Supplement: S5 Fig — (A-J) Venn diagrams comparing genes that have at least one MACS2 peak for each chromatin modification, across time points and within sex. For each Venn diagram category, the number of genes and the proportion of the total genes (in parentheses) in each panel is shown. The histone modification is indicated on the left (activating in purple, repressive in black). The legend for each time point is on the top left and male and female data sets are indicated at the top. All MACS2 peaks were called on pooled biological replicates (n = 3–4) and identified as enriched relative to matched input controls. (K) Venn diagrams for genes with bivalent promoter regions (containing both H3K4me3 and H3K27me3) at each time point in fru P1 neurons in males (left) and females (right). MACS2 peaks and genes from Venn diagram analyses are listed in S1–S3 Data, S5–S7 Data and S2 Table. (PDF) [file pgen.1009240.s005.pdf]

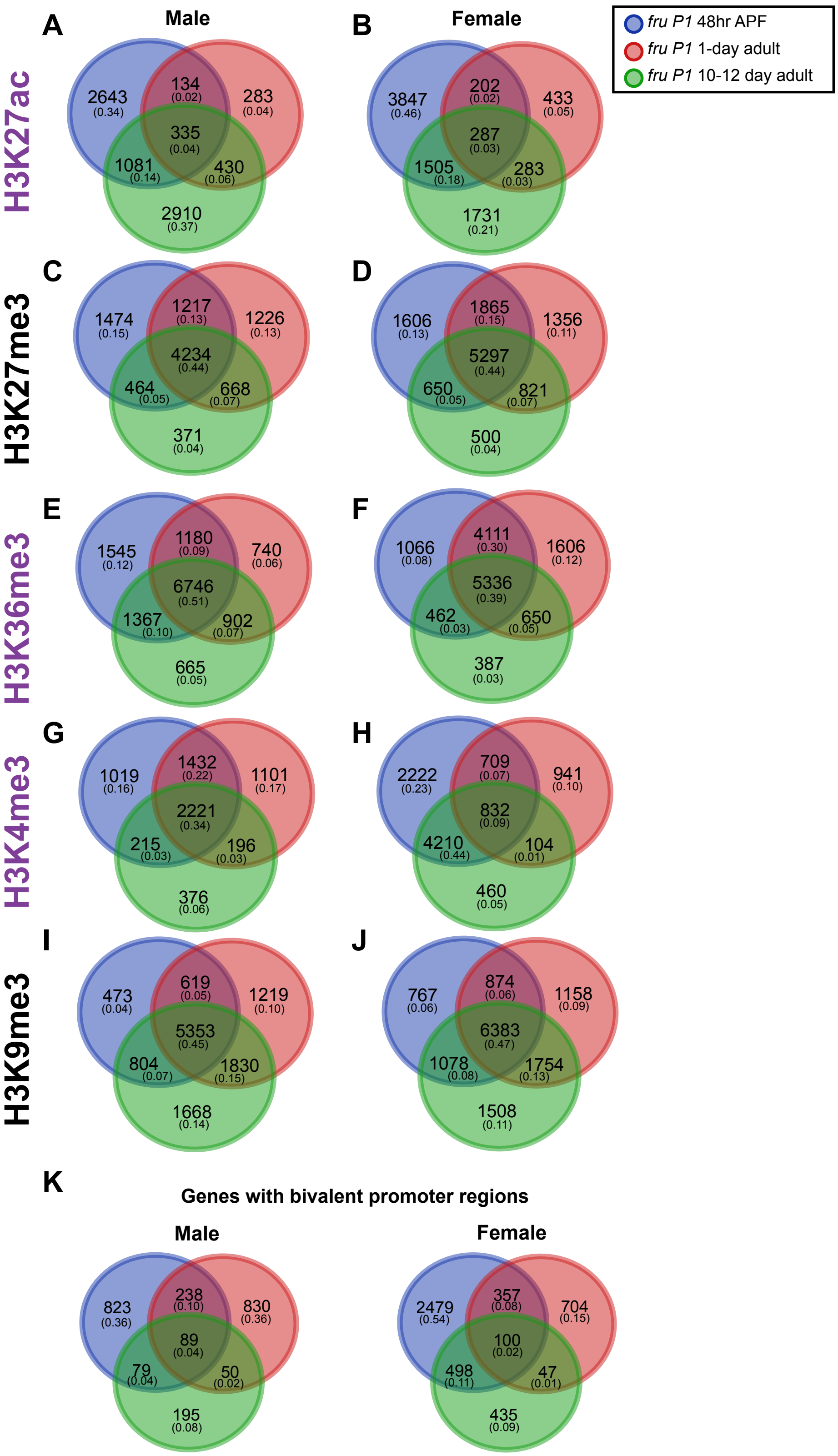
A
Male
B
Female
fru P1 48hr APF
fru P1 1-day adult
fru P1 10-12 day adult
H3K27ac
2643 (0.34)
134 (0.02)
283 (0.04)
335 (0.04)
1081 (0.14)
430 (0.06)
2910 (0.37)
3847 (0.46)
202 (0.02)
433 (0.05)
287 (0.03)
1505 (0.18)
283 (0.03)
1731 (0.21)
C
D
H3K27me3
1474 (0.15)
1217 (0.13)
1226 (0.13)
4234 (0.44)
464 (0.05)
668 (0.07)
371 (0.04)
1606 (0.13)
1865 (0.15)
1356 (0.11)
5297 (0.44)
650 (0.05)
821 (0.07)
500 (0.04)
E
F
H3K36me3
1545 (0.12)
1180 (0.09)
740 (0.06)
6746 (0.51)
1367 (0.10)
902 (0.07)
665 (0.05)
1066 (0.08)
4111 (0.30)
1606 (0.12)
5336 (0.39)
462 (0.03)
650 (0.05)
387 (0.03)
G
H
H3K4me3
1019 (0.16)
1432 (0.22)
1101 (0.17)
2221 (0.34)
215 (0.03)
196 (0.03)
376 (0.06)
2222 (0.23)
709 (0.07)
941 (0.10)
832 (0.09)
4210 (0.44)
104 (0.01)
460 (0.05)
I
J
H3K9me3
473 (0.04)
619 (0.05)
1219 (0.10)
5353 (0.45)
804 (0.07)
1830 (0.15)
1668 (0.14)
767 (0.06)
874 (0.06)
1158 (0.09)
6383 (0.47)
1078 (0.08)
1754 (0.13)
1508 (0.11)
K
Genes with bivalent promoter regions
Male
Female
823 (0.36)
238 (0.10)
830 (0.36)
89 (0.04)
79 (0.04)
50 (0.02)
195 (0.08)
2479 (0.54)
357 (0.08)
704 (0.15)
100 (0.02)
498 (0.11)
47 (0.01)
435 (0.09)

S5 Fig. Overlap of genes containing MACS2 peaks and genes with bivalent promoters across time points in *fru P1* neurons. **(A-J)** Venn diagrams comparing genes that have at least one MACS2 peak for each chromatin modification, across time points and within sex. For each Venn diagram category, the number of genes and the proportion of the total genes (in parentheses) in each panel is shown. The histone modification is indicated on the left (activating in purple, repressive in black). The legend for each time point is on the top left and male and female data sets are indicated at the top. All MACS2 peaks were called on pooled biological replicates (n=3-4) and identified as enriched relative to matched input controls. (**K**) Venn diagrams for genes with bivalent promoter regions (containing both H3K4me3 and H3K27me3) at each time point in *fru P1* neurons in males (left) and females (right). MACS2 peaks and genes from Venn diagram analyses are listed in **S1-S3 Data, S5-S7 Data** and **S2 Table**.
