# Supplementary material for: Analysis of cell-type-specific chromatin modifications and gene expression in Drosophila neurons that direct reproductive behavior
Source: PLoS Genet. 2021 Apr 26;17(4):e1009240. doi: 10.1371/journal.pgen.1009240 (PMC8102012; doi:10.1371/journal.pgen.1009240)
Supplement: S6 Fig — (A-B) Gene Ontology (GO) enrichment analysis for genes with modifications that persist across all three time points in fru P1 neurons in (A) males and (B) females (centers of Venn diagrams in S5 Fig). The GO categories are molecular function, biological process, and cellular component. The GO terms shown in the plots are the top ten most significantly enriched terms for each list (non-redundant shown; Benjamini-Hochberg, p<0.05). The size of each dot indicates the number of genes (count) and the color indicates the p value (p.adjust). The GO information is in S3 Table and gene lists are in S2 Table. (PDF) [file pgen.1009240.s006.pdf]

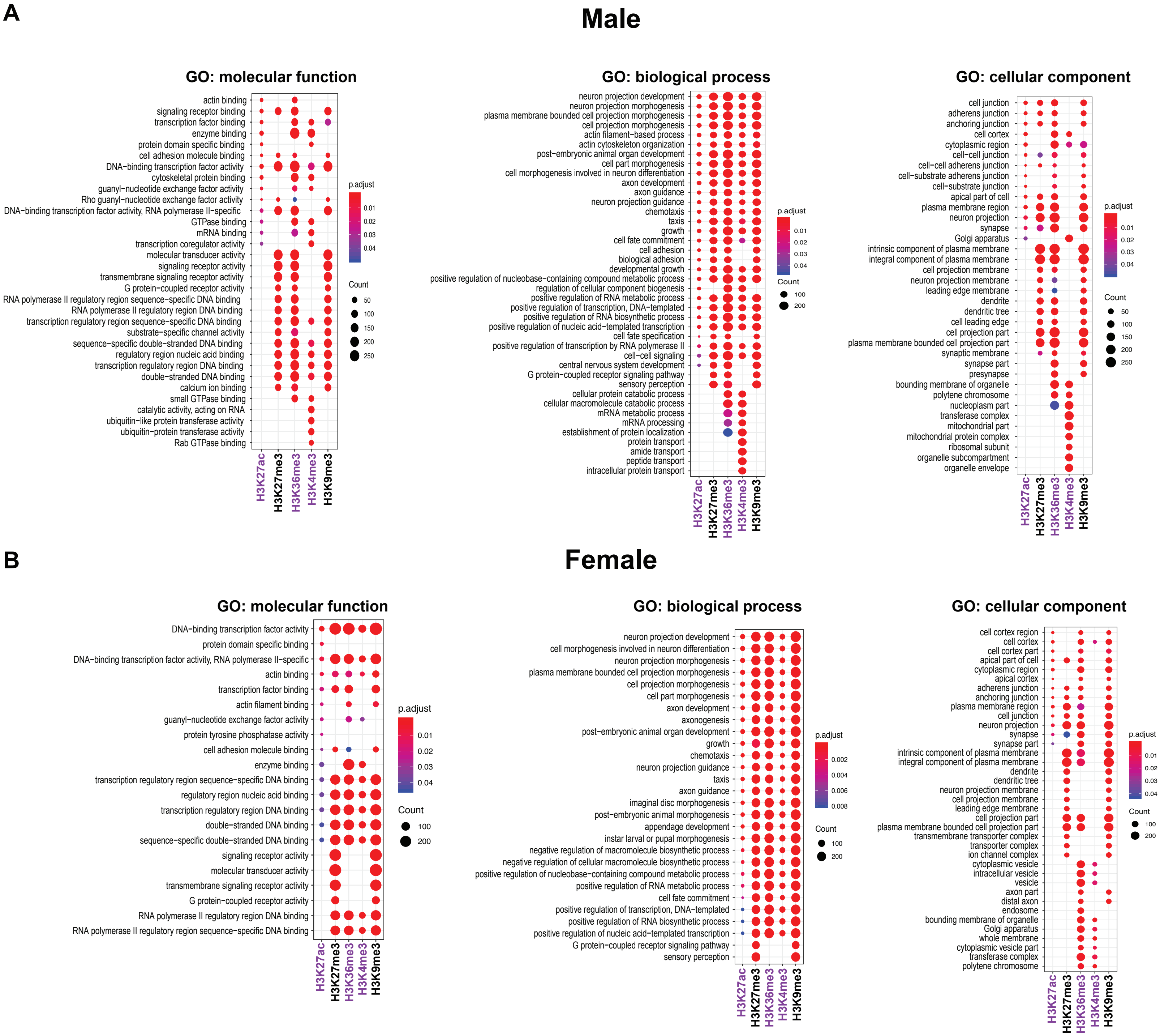
A
Male
GO: molecular function
actin binding
signaling receptor binding
transcription factor binding
enzyme binding
protein domain specific binding
cell adhesion molecule binding
DNA−binding transcription factor activity
cytoskeletal protein binding
guanyl−nucleotide exchange factor activity
Rho guanyl−nucleotide exchange factor activity
DNA−binding transcription factor activity, RNA polymerase II−specific
GTPase binding
mRNA binding
transcription coregulator activity
molecular transducer activity
signaling receptor activity
transmembrane signaling receptor activity
G protein−coupled receptor activity
RNA polymerase II regulatory region sequence−specific DNA binding
RNA polymerase II regulatory region DNA binding
transcription regulatory region sequence−specific DNA binding
substrate−specific channel activity
sequence−specific double−stranded DNA binding
regulatory region nucleic acid binding
transcription regulatory region DNA binding
double−stranded DNA binding
calcium ion binding
small GTPase binding
catalytic activity, acting on RNA
ubiquitin−like protein transferase activity
ubiquitin−protein transferase activity
Rab GTPase binding
H3K27ac
H3K27me3
H3K36me3
H3K4me3
H3K9me3
p.adjust
0.01
0.02
0.03
0.04
Count
50
100
150
200
250
GO: biological process
neuron projection development
neuron projection morphogenesis
plasma membrane bounded cell projection morphogenesis
cell projection morphogenesis
actin filament−based process
actin cytoskeleton organization
post−embryonic animal organ development
cell part morphogenesis
cell morphogenesis involved in neuron differentiation
axon development
axon guidance
neuron projection guidance
chemotaxis
taxis
growth
cell fate commitment
cell adhesion
biological adhesion
developmental growth
positive regulation of nucleobase−containing compound metabolic process
regulation of cellular component biogenesis
positive regulation of RNA metabolic process
positive regulation of transcription, DNA−templated
positive regulation of RNA biosynthetic process
positive regulation of nucleic acid−templated transcription
cell fate specification
positive regulation of transcription by RNA polymerase II
cell−cell signaling
central nervous system development
G protein−coupled receptor signaling pathway
sensory perception
cellular protein catabolic process
cellular macromolecule catabolic process
mRNA metabolic process
mRNA processing
establishment of protein localization
protein transport
amide transport
peptide transport
intracellular protein transport
H3K27ac
H3K27me3
H3K36me3
H3K4me3
H3K9me3
p.adjust
0.01
0.02
0.03
0.04
Count
100
200
GO: cellular component
cell junction
adherens junction
anchoring junction
cell cortex
cytoplasmic region
cell−cell junction
cell−cell adherens junction
cell−substrate adherens junction
cell−substrate junction
apical part of cell
plasma membrane region
neuron projection
synapse
Golgi apparatus
intrinsic component of plasma membrane
integral component of plasma membrane
cell projection membrane
neuron projection membrane
leading edge membrane
dendrite
dendritic tree
cell leading edge
cell projection part
plasma membrane bounded cell projection part
synaptic membrane
synapse part
presynapse
bounding membrane of organelle
polytene chromosome
nucleoplasm part
transferase complex
mitochondrial part
mitochondrial protein complex
ribosomal subunit
organelle subcompartment
organelle envelope
H3K27ac
H3K27me3
H3K36me3
H3K4me3
H3K9me3
p.adjust
0.01
0.02
0.03
0.04
Count
50
100
150
200
250
B
Female
GO: molecular function
DNA−binding transcription factor activity
protein domain specific binding
DNA−binding transcription factor activity, RNA polymerase II−specific
actin binding
transcription factor binding
actin filament binding
guanyl−nucleotide exchange factor activity
protein tyrosine phosphatase activity
cell adhesion molecule binding
enzyme binding
transcription regulatory region sequence−specific DNA binding
regulatory region nucleic acid binding
transcription regulatory region DNA binding
double−stranded DNA binding
sequence−specific double−stranded DNA binding
signaling receptor activity
molecular transducer activity
transmembrane signaling receptor activity
G protein−coupled receptor activity
RNA polymerase II regulatory region DNA binding
RNA polymerase II regulatory region sequence−specific DNA binding
H3K27ac
H3K27me3
H3K36me3
H3K4me3
H3K9me3
p.adjust
0.01
0.02
0.03
0.04
Count
100
200
GO: biological process
neuron projection development
cell morphogenesis involved in neuron differentiation
neuron projection morphogenesis
plasma membrane bounded cell projection morphogenesis
cell projection morphogenesis
cell part morphogenesis
axon development
axonogenesis
post−embryonic animal organ development
growth
chemotaxis
neuron projection guidance
taxis
axon guidance
imaginal disc morphogenesis
post−embryonic animal morphogenesis
appendage development
instar larval or pupal morphogenesis
negative regulation of macromolecule biosynthetic process
negative regulation of cellular macromolecule biosynthetic process
positive regulation of nucleobase−containing compound metabolic process
positive regulation of RNA metabolic process
cell fate commitment
positive regulation of transcription, DNA−templated
positive regulation of RNA biosynthetic process
positive regulation of nucleic acid−templated transcription
G protein−coupled receptor signaling pathway
sensory perception
H3K27ac
H3K27me3
H3K36me3
H3K4me3
H3K9me3
p.adjust
0.002
0.004
0.006
0.008
Count
100
200
GO: cellular component
cell cortex region
cell cortex
cell cortex part
apical part of cell
cytoplasmic region
apical cortex
adherens junction
anchoring junction
plasma membrane region
cell junction
neuron projection
synapse
synapse part
intrinsic component of plasma membrane
integral component of plasma membrane
dendrite
dendritic tree
neuron projection membrane
cell projection membrane
leading edge membrane
cell projection part
plasma membrane bounded cell projection part
transmembrane transporter complex
transporter complex
ion channel complex
cytoplasmic vesicle
intracellular vesicle
vesicle
axon part
distal axon
endosome
bounding membrane of organelle
Golgi apparatus
whole membrane
cytoplasmic vesicle part
transferase complex
polytene chromosome
H3K27ac
H3K27me3
H3K36me3
H3K4me3
H3K9me3
p.adjust
0.01
0.02
0.03
0.04
Count
100
200

S6 Fig. GO for genes with modifications that persist across all three time points in *fru P1* neurons. (**A-B**) Gene Ontology (GO) enrichment analysis for genes with modifications that persist across all three time points in *fru P1* neurons in (**A**) males and (**B**) females (centers of Venn diagrams in **S5 Fig**). The GO categories are molecular function, biological process, and cellular component. The GO terms shown in the plots are the top ten most significantly enriched terms for each list (non-redundant shown; Benjamini-Hochberg, $p<0.05$). The size of each dot indicates the number of genes (count) and the color indicates the p value (p.adjust). The GO information is in **S3 Table** and gene lists are in **S2 Table**.
