# Supplementary material for: Analysis of cell-type-specific chromatin modifications and gene expression in Drosophila neurons that direct reproductive behavior
Source: PLoS Genet. 2021 Apr 26;17(4):e1009240. doi: 10.1371/journal.pgen.1009240 (PMC8102012; doi:10.1371/journal.pgen.1009240)
Supplement: S7 Fig — (A) Average read coverage in 48hr APF TRAP and (B) 10–12 day adult TRAP for males (blue) and females (red) across the fruitless locus. The 5’ end of fruitless is located on the right and transcription runs in the direction of the arrows on the gene structure (right to left). (C) Sex-biased TRAP genes were identified by comparing expression between the male and female TRAP samples. For a gene to be considered sex-biased in the TRAP comparison, at least one exon needed to be more highly expressed in the TRAP sample of one sex (FDR<0.2). Comparison of male-biased TRAP genes (teal) and female-biased TRAP genes (purple) for all time points examined. For each Venn diagram category, the number of genes and the proportion of the total genes (in parentheses) in each panel is shown. (D-E) Venn diagrams comparing sex-biased TRAP genes across time points within each sex. (F) Upset intersection plot of sex-biased TRAP genes from 48hr APF (blue), 1-day adult (red) [42] and 10–12 day adult (green) time points [71]. n = 4–5 biological replicates per condition. (G) Gene Ontology (GO) enrichment analysis for sex-biased TRAP genes. The GO categories are molecular function, biological process, and cellular component. The GO terms shown in the plots are the top ten most significantly enriched terms for each list (non-redundant shown; Benjamini-Hochberg, p<0.05). The size of each dot indicates the number of genes (count) and the color indicates the p value (p.adjust). The GO information is in S3 Table and gene lists are in S9 and S10 Tables. (PDF) [file pgen.1009240.s007.pdf]

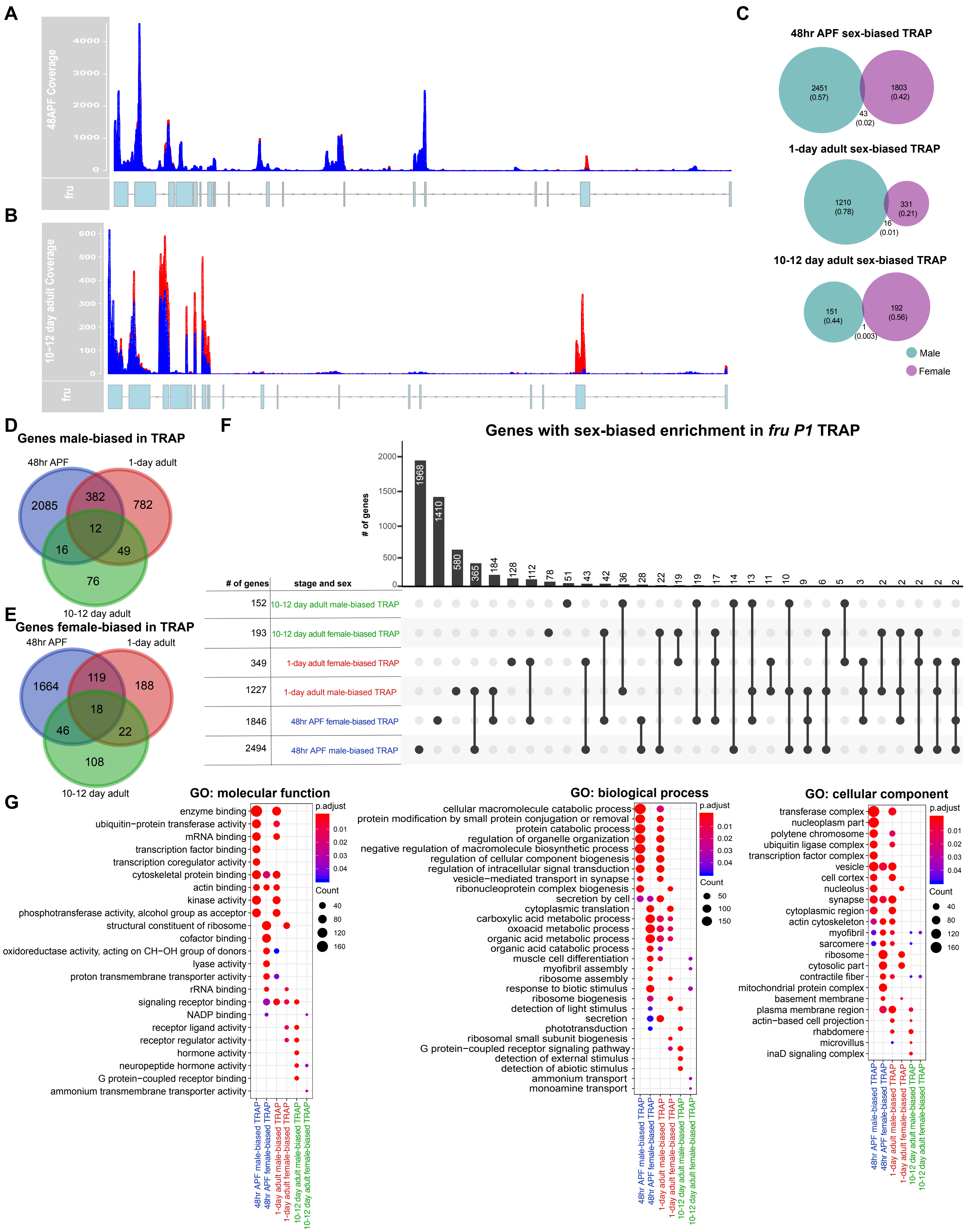
A
48APF Coverage
fru
B
10-12 day adult Coverage
fru
C
48hr APF sex-biased TRAP
2451 (0.57)
1803 (0.42)
43 (0.02)
1-day adult sex-biased TRAP
1210 (0.78)
331 (0.21)
16 (0.01)
10-12 day adult sex-biased TRAP
151 (0.44)
192 (0.56)
1 (0.003)
Male
Female
D
Genes male-biased in TRAP
48hr APF
1-day adult
10-12 day adult
E
Genes female-biased in TRAP
F
Genes with sex-biased enrichment in fru P1 TRAP
# of genes
# of genes
stage and sex
152 10-12 day adult male-biased TRAP
193 10-12 day adult female-biased TRAP
349 1-day adult female-biased TRAP
1227 1-day adult male-biased TRAP
1846 48hr APF female-biased TRAP
2494 48hr APF male-biased TRAP
G
GO: molecular function
GO: biological process
GO: cellular component
p.adjust
Count

S7 Fig. TRAP sequencing read coverage of *fruitless* in 48hr APF and 10-12 day data sets and analysis of sex-biased TRAP genes. (A) Average read coverage in 48hr APF TRAP and (**B**) 10-12 day adult TRAP for males (blue) and females (red) across the *fruitless* locus. The 5' end of *fruitless* is located on the right and transcription runs in the direction of the arrows on the gene structure (right to left). **(C)** Sex-biased TRAP genes were identified by comparing expression between the male and female TRAP samples. For a gene to be considered sex-biased in the TRAP comparison, at least one exon needed to be more highly expressed in the TRAP sample of one sex (FDR<0.2). Comparison of male-biased TRAP genes (teal) and female-biased TRAP genes (purple) for all time points examined. For each Venn diagram category, the number of genes and the proportion of the total genes (in parentheses) in each panel is shown. (**D-E**) Venn diagrams comparing sex-biased TRAP genes across time points within each sex. (**F**) Upset intersection plot of sex-biased TRAP genes from 48hr APF (blue), 1-day adult (red) [42] and 10-12 day adult (green) time points [71]. n=4-5 biological replicates per condition. (**G**) Gene Ontology (GO) enrichment analysis for sex-biased TRAP genes. The GO categories are molecular function, biological process, and cellular component. The GO terms shown in the plots are the top ten most significantly enriched terms for each list (non-redundant shown; Benjamini-Hochberg, p<0.05). The size of each dot indicates the number of genes (count) and the color indicates the p value (p.adjust). The GO information is in **S3 Table** and gene lists are in **S9-S10 Tables**.
