# Supplementary material for: Analysis of cell-type-specific chromatin modifications and gene expression in Drosophila neurons that direct reproductive behavior
Source: PLoS Genet. 2021 Apr 26;17(4):e1009240. doi: 10.1371/journal.pgen.1009240 (PMC8102012; doi:10.1371/journal.pgen.1009240)
Supplement: S8 Fig — For detailed description of heatmaps see Fig 3. Heatmaps for elav chromatin data from 1-day adults based on elav TRAP data enrichments [41]. (A-B) Heatmap for genes that are TRAP-enriched in 1-day adult elav neurons. (C-D) Heatmap for genes enriched in total mRNA. For each cluster of genes, the average fold-enrichment (Avg FC) of gene expression, indicated on the left (TRAP-enrichment), was calculated. For each cluster of genes, the average expression is indicated on the right for the TRAP and total mRNA data. Average fold-enrichment in gene expression and average expression in fragments per kilobase per million (FPKM) was calculated per cluster. The average was from expression data for genes that were significantly enriched in elav neurons (TRAP) from both sexes, or whole head tissue (total mRNA), averaged over both sexes (FDR<0.2; [41]). Gene lists for each cluster are provided in S4 Table. (PDF) [file pgen.1009240.s008.pdf]

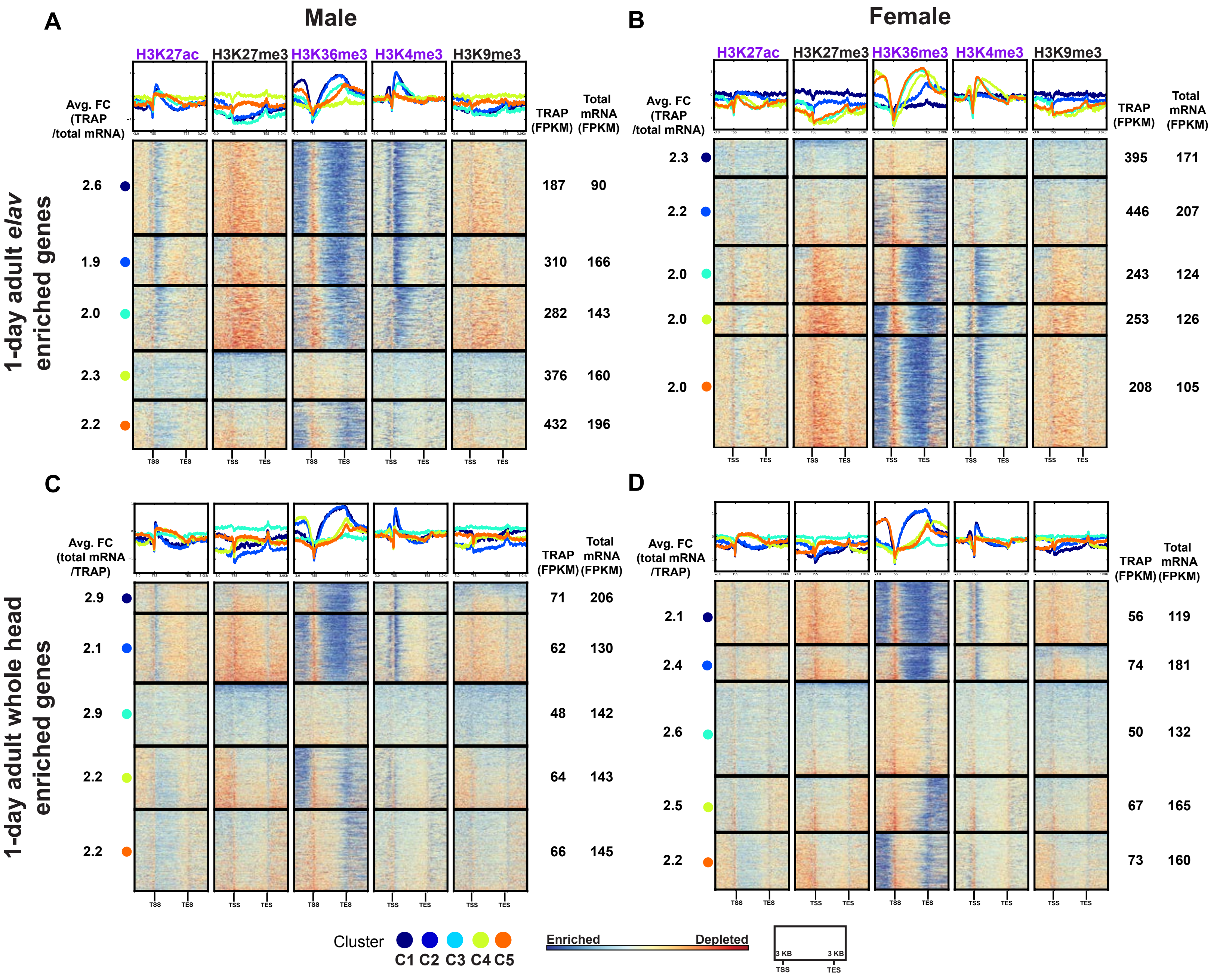

A
Male
B
Female
H3K27ac
H3K27me3
H3K36me3
H3K4me3
H3K9me3
Avg. FC (TRAP /total mRNA)
TRAP (FPKM)
Total mRNA (FPKM)
1-day adult elav enriched genes
2.6 187 90
1.9 310 166
2.0 282 143
2.3 376 160
2.2 432 196
2.3 395 171
2.2 446 207
2.0 243 124
2.0 253 126
2.0 208 105
TSS
TES
C
D
Avg. FC (total mRNA /TRAP)
1-day adult whole head enriched genes
2.9 71 206
2.1 62 130
2.9 48 142
2.2 64 143
2.2 66 145
2.1 56 119
2.4 74 181
2.6 50 132
2.5 67 165
2.2 73 160
Cluster
C1 C2 C3 C4 C5
Enriched
Depleted
3 KB
3 KB

S8 Fig. Hierarchical clustering of histone modification distributions for elav TRAP-enriched genes. For detailed description of heatmaps see **Fig 3**. Heatmaps for *elav* chromatin data from 1-day adults based on *elav* TRAP data enrichments [41]. **(A-B)** Heatmap for genes that are TRAP-enriched in 1-day adult *elav* neurons. **(C-D)** Heatmap for genes enriched in total mRNA. For each cluster of genes, the average fold-enrichment (Avg FC) of gene expression, indicated on the left (TRAP-enrichment), was calculated. For each cluster of genes, the average expression is indicated on the right for the TRAP and total mRNA data. Average fold-enrichment in gene expression and average expression in fragments per kilobase per million (FPKM) was calculated per cluster. The average was from expression data for genes that were significantly enriched in *elav* neurons (TRAP) from both sexes, or whole head tissue (total mRNA), averaged over both sexes (FDR<0.2; [41]). Gene lists for each cluster are provided in **S4 Table**.
