# Supplementary material for: Analysis of cell-type-specific chromatin modifications and gene expression in Drosophila neurons that direct reproductive behavior
Source: PLoS Genet. 2021 Apr 26;17(4):e1009240. doi: 10.1371/journal.pgen.1009240 (PMC8102012; doi:10.1371/journal.pgen.1009240)
Supplement: S9 Fig — Hierarchal clustering reveals shared combinations of histone modifications for total mRNA-enriched genes relative to TRAP, generated using deepTools. For detailed description of heatmaps see Fig 3. Heatmaps for fru P1 chromatin data from 48hr APF (A-B), 1-day adults (C-D), and 10–12 day adults (E-F). Gene lists for each cluster are provided in S4 Table. For each cluster of genes, the average fold-enrichment (Avg FC) of gene expression is indicated on the left (total mRNA-enrichment), calculated at the exon level. For each cluster of genes, the average expression is indicated on the right for the TRAP and total mRNA data, calculated at the exon level. The 48hr APF and 10–12 day adult gene expression levels are provided as reads per million (RPM). The 1-day adult time point gene expression levels are provided as reads per kilobase per million (RPKM) [42]. For TRAP and total mRNA expression data see S6–S8 Tables. (PDF) [file pgen.1009240.s009.pdf]

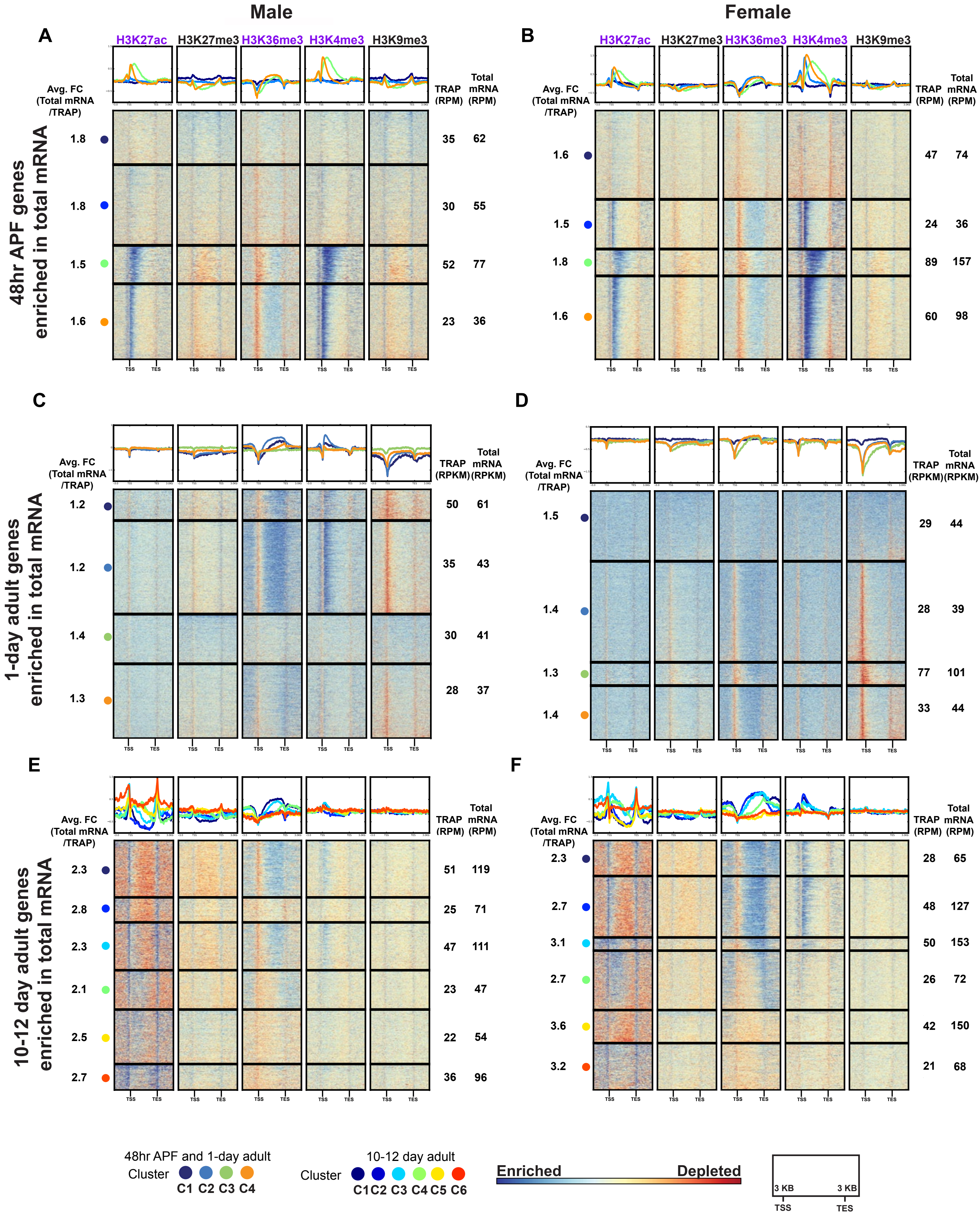

Male
Female
A
B
C
D
E
F
H3K27ac
H3K27me3
H3K36me3
H3K4me3
H3K9me3
Avg. FC (Total mRNA /TRAP)
TRAP (RPM)
Total mRNA (RPM)
TRAP (RPKM)
Total mRNA (RPKM)
48hr APF genes enriched in total mRNA
1-day adult genes enriched in total mRNA
10-12 day adult genes enriched in total mRNA
1.8 35 62
1.8 30 55
1.5 52 77
1.6 23 36
1.6 47 74
1.5 24 36
1.8 89 157
1.6 60 98
1.2 50 61
1.2 35 43
1.4 30 41
1.3 28 37
1.5 29 44
1.4 28 39
1.3 77 101
1.4 33 44
2.3 51 119
2.8 25 71
2.3 47 111
2.1 23 47
2.5 22 54
2.7 36 96
2.3 28 65
2.7 48 127
3.1 50 153
2.7 26 72
3.6 42 150
3.2 21 68
TSS
TES
48hr APF and 1-day adult
Cluster
C1 C2 C3 C4
10-12 day adult
Cluster
C1 C2 C3 C4 C5 C6
Enriched
Depleted
3 KB
3 KB
TSS
TES

S9 Fig. Hierarchical clustering of histone modification distributions for total mRNA-enriched genes. Hierarchal clustering reveals shared combinations of histone modifications for total mRNA-enriched genes relative to TRAP, generated using deepTools. For detailed description of heatmaps see **Fig 3**. Heatmaps for *fru P1* chromatin data from 48hr APF **(A-B)**, 1-day adults **(C-D)**, and 10-12 day adults **(E-F)**. Gene lists for each cluster are provided in **S4 Table**. For each cluster of genes, the average fold-enrichment (Avg FC) of gene expression is indicated on the left (total mRNA-enrichment), calculated at the exon level. For each cluster of genes, the average expression is indicated on the right for the TRAP and total mRNA data, calculated at the exon level. The 48hr APF and 10-12 day adult gene expression levels are provided as reads per million (RPM). The 1-day adult time point gene expression levels are provided as reads per kilobase per million (RPKM) [42]. For TRAP and total mRNA expression data see **S6-S8 Tables**.
