# Supplementary material for: Analysis of cell-type-specific chromatin modifications and gene expression in Drosophila neurons that direct reproductive behavior
Source: PLoS Genet. 2021 Apr 26;17(4):e1009240. doi: 10.1371/journal.pgen.1009240 (PMC8102012; doi:10.1371/journal.pgen.1009240)
Supplement: S10 Fig — Hierarchal clustering reveals shared combinations of histone modifications for genes detected in expression data sets, generated using deepTools. For detailed description of heatmaps see Fig 3. Heatmaps for fru P1 chromatin data from 48hr APF (A-B), 1-day adults (C-D), and 10–12 day adults (E-F). A gene was considered detected in our 48hr APF or 10–12 day adult TRAP libraries if one or more exons were detected based on our initial filtering criteria (see Materials and Methods). Genes detected in 1-day adult TRAP were those identified in [42]. Gene lists for each cluster are provided in S4 Table. (PDF) [file pgen.1009240.s010.pdf]

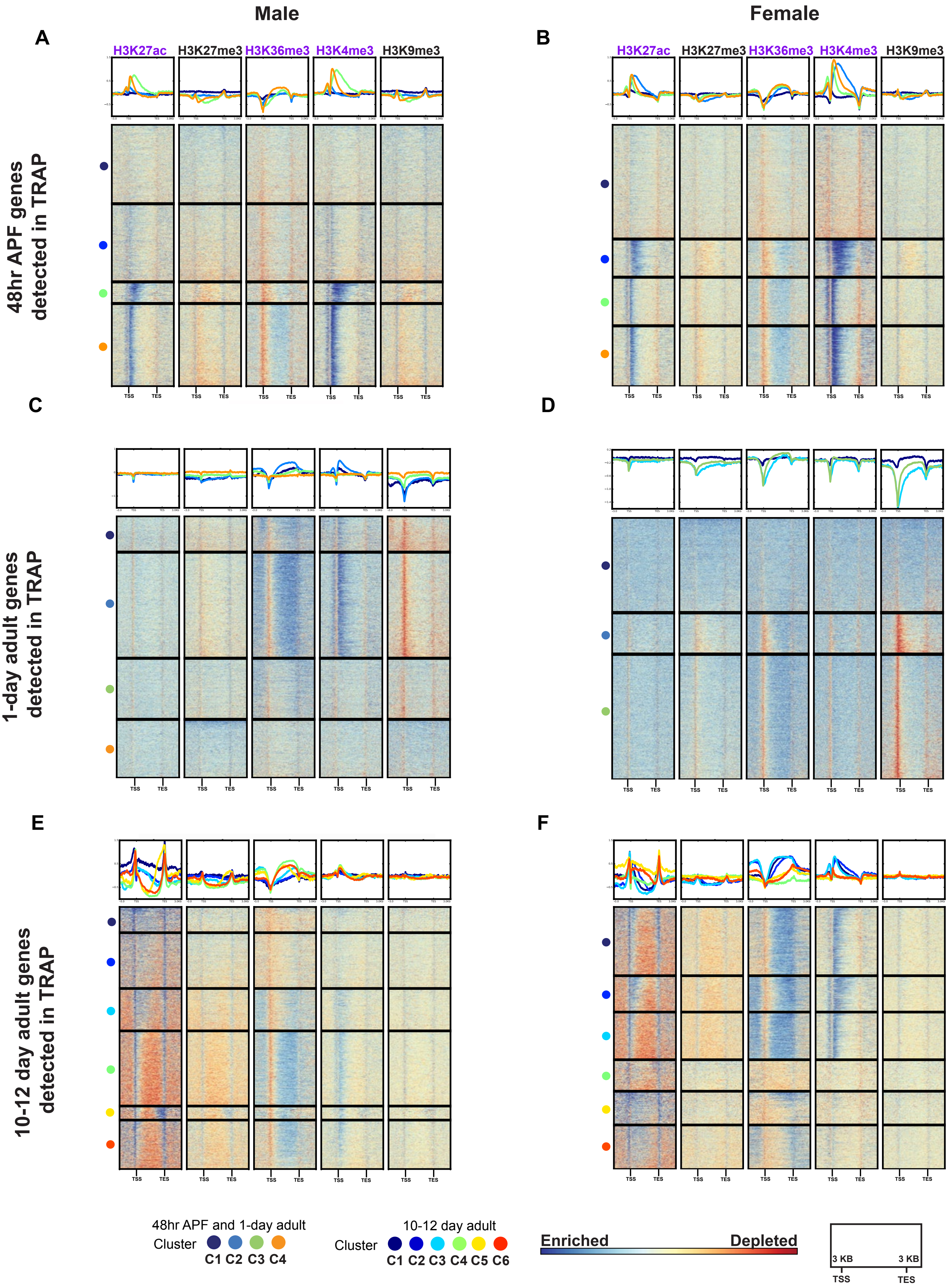

Male
Female
A
B
C
D
E
F
H3K27ac
H3K27me3
H3K36me3
H3K4me3
H3K9me3
48hr APF genes detected in TRAP
1-day adult genes detected in TRAP
10-12 day adult genes detected in TRAP
TSS
TES
48hr APF and 1-day adult
Cluster
C1 C2 C3 C4
10-12 day adult
Cluster
C1 C2 C3 C4 C5 C6
Enriched
Depleted
3 KB
3 KB
TSS
TES

S10 Fig. Hierarchical clustering of histone modification distributions for TRAP-detected genes. Hierarchal clustering reveals shared combinations of histone modifications for genes detected in expression data sets, generated using deepTools. For detailed description of heatmaps see **Fig 3**. Heatmaps for *fru P1* chromatin data from 48hr APF **(A-B)**, 1-day adults **(C-D)**, and 10-12 day adults **(E-F)**. A gene was considered detected in our 48hr APF or 10-12 day adult TRAP libraries if one or more exons were detected based on our initial filtering criteria (see Materials and Methods). Genes detected in 1-day adult TRAP were those identified in [42]. Gene lists for each cluster are provided in **S4 Table**.
