# Supplementary material for: Analysis of cell-type-specific chromatin modifications and gene expression in Drosophila neurons that direct reproductive behavior
Source: PLoS Genet. 2021 Apr 26;17(4):e1009240. doi: 10.1371/journal.pgen.1009240 (PMC8102012; doi:10.1371/journal.pgen.1009240)
Supplement: S11 Fig — (A) Venn diagrams showing the overlap between fru P1 SE-containing genes identified on autosomes across time points for males (left) and females (right). (B) Percentages of SE-containing genes for each stage and neuron type (fru P1 or elav) that are male-specific, female-specific or occur in both sexes. (PDF) [file pgen.1009240.s011.pdf]

Autosomes only

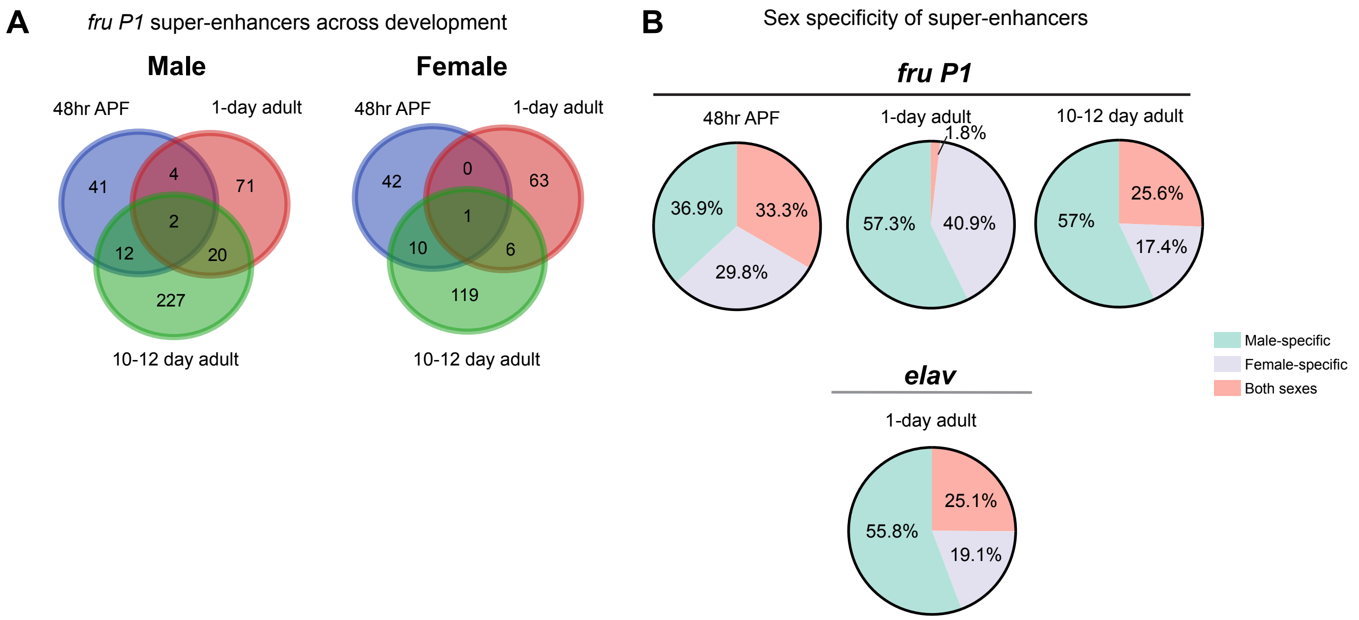

S11 Fig. Super-enhancers identified based on H3K27ac peaks in *fru P1*- and *elav* neuron chromatin data sets for autosomes. **(A)** Venn diagrams showing the overlap between *fru P1* SE-containing genes identified on autosomes across time points for males (left) and females (right). **(B)** Percentages of SE-containing genes for each stage and neuron type (*fru P1* or *elav*) that are male-specific, female-specific or occur in both sexes.
